# Archaeological Soybean (*Glycine max*) in East Asia: Does Size Matter?

**DOI:** 10.1371/journal.pone.0026720

**Published:** 2011-11-04

**Authors:** Gyoung-Ah Lee, Gary W. Crawford, Li Liu, Yuka Sasaki, Xuexiang Chen

**Affiliations:** 1 Department of Anthropology, University of Oregon, Eugene, Oregon, United States of America; 2 Department of Anthropology, University of Toronto Mississauga, Mississauga, Ontario, Canada; 3 East Asian Languages and Cultures, Stanford Archaeology Center, Stanford University, Stanford, California, United States of America; 4 Paleo Labo Co., Ltd., Toda, Japan; 5 Department of Archaeology, Shandong University, Jinan, China; The Pennsylvania State University, United States of America

## Abstract

The recently acquired archaeological record for soybean from Japan, China and Korea is shedding light on the context in which this important economic plant became associated with people and was domesticated. This paper examines archaeological (charred) soybean seed size variation to determine what insight can be gained from a comprehensive comparison of 949 specimens from 22 sites. Seed length alone appears to represent seed size change through time, although the length×width×thickness product has the potential to provide better size change resolution. A widespread early association of small seeded soybean is as old as 9000–8600 cal BP in northern China and 7000 cal BP in Japan. Direct AMS radiocarbon dates on charred soybean seeds indicate selection resulted in large seed sizes in Japan by 5000 cal BP (Middle Jomon) and in Korea by 3000 cal BP (Early Mumun). Soybean seeds recovered in China from the Shang through Han periods are similar in length to the large Korean and Japanese specimens, but the overall size of the large Middle and Late Jomon, Early Mumun through Three Kingdom seeds is significantly larger than any of the Chinese specimens. The archaeological record appears to disconfirm the hypothesis of a single domestication of soybean and supports the view informed by recent phyologenetic research that soybean was domesticated in several locations in East Asia.

## Introduction

**Figure 1:**
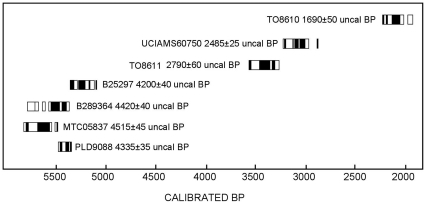
Direct dates on charred soybean seeds. Black bars indicate 2σ range; white box indicates 1σ range of the calibrated dates. PLD9088: Shimoyakebe, Japan; MTC05837: Shimoyakebe, Japan; B289364: Dahecun, China; B25927: Pyeonggeodong, South Korea; TO8611: Okbang 1/9 (Nam River), South Korea; UCIAMS60750: Daundong, South Korea; TO8610: Three Kingdom, South Korea. B, Beta Analytic, USA; PLD, Paleo Labo Co., Ltd., Japan; MTC, Research Center of Nuclear Science and Technology, University of Tokyo, Japan; TO, University of Toronto Isotrace Laboratory, Canada; UCIAMS, University of California Irvine Keck Carbon Cycle AMS Facility, USA. All the materials dated are charred. Conventional dates were calibrated with Calib 6.0 using the Intcal 09 curve [8], [9], [10].

Soybean (*Glycine max* subsp. *max*) is the world's foremost oilseed source and the primary source of protein for chickens and pigs [1] and ranks seventh among world crops by tonnage harvested [2]. Despite the importance of the crop to the world economy, how soybean came to be a crucial resource and ultimately a domesticated plant has not been enlightened by the archaeological record. Instead, the history of soybean has been informed by phylogenetics and historical documents indicating that soybean was domesticated in East Asia and that it became an important crop by the Zhou Dynasty (ca. 2500 BP) in China. However, the details of where, when, and under what circumstances soybean developed a close relationship with people are poorly understood. One oft-cited (e.g. [3], [4]) but archaeologically unsubstantiated source claims that soybean was domesticated “in ancient China perhaps 3000 to 5000 years ago” [5] leading many botanists and historians to believe that the problem of soybean domestication is resolved. Yet Carter *et al.* [5] clarify that the question is still open. In the 2000s two of us [6] documented the first unambiguously domesticated soybean in East Asia from the Daundong and Nam River (Okbang 1/9) sites in South Korea, with two AMS-dates on soybean from ca. 2720–2380 BP (Figure 1). This suggested that the hypothesis that soybean was domesticated somewhere in Northeast Asia (potentially in Korea) had merit [7].

**Figure 2:**
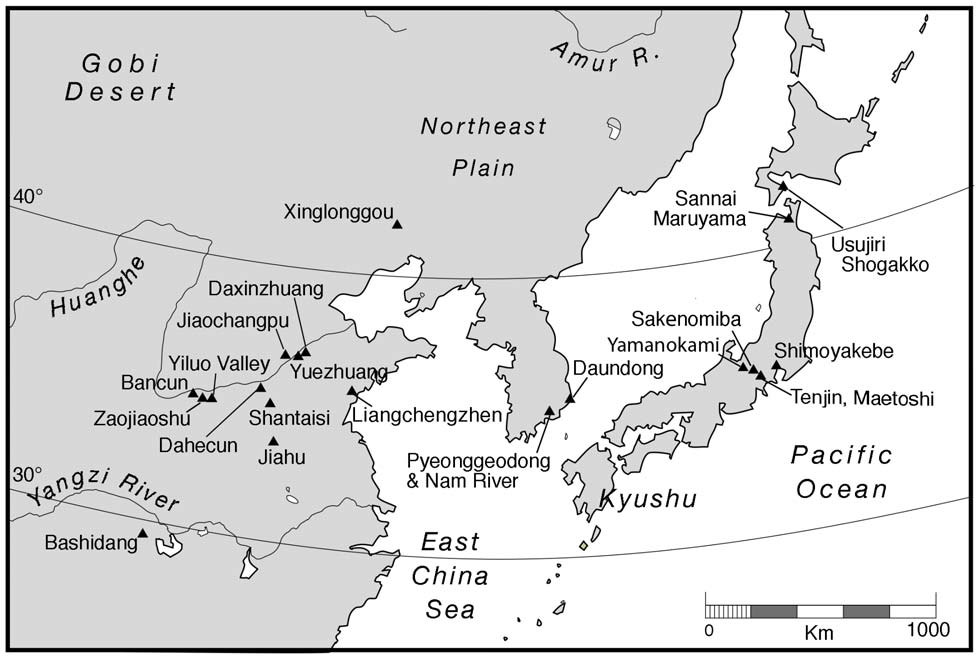
Locations of Sites discussed in the text.

**Figure 3:**
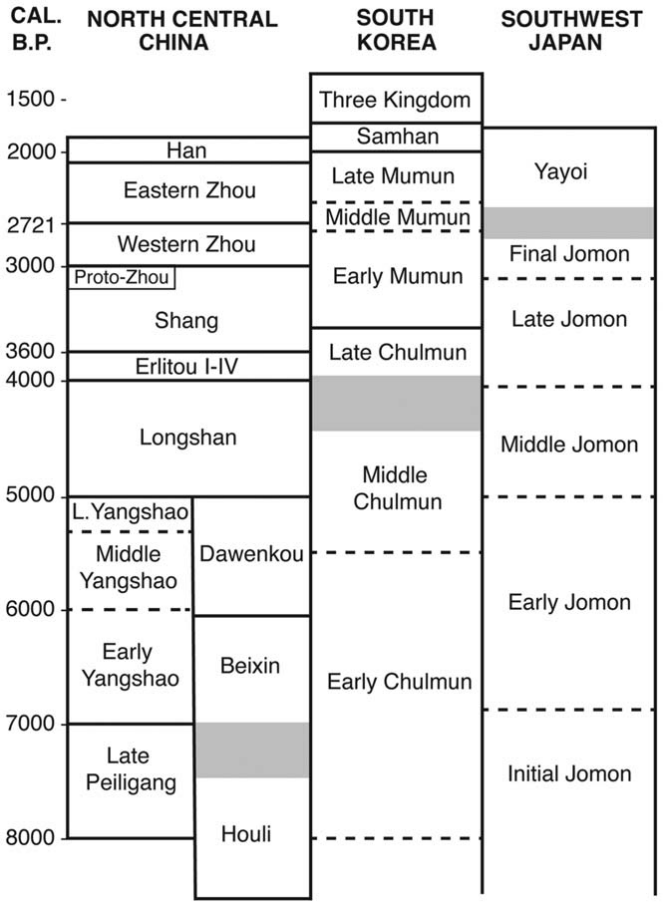
Chronology relevant to the regions discussed in paper. The Proto-Zhou period represents non-Shang culture in western Shaanxi that is contemporaneous with Late Shang centered in Henan.

However, since 2003 archaeological research has brought to light a more extensive archaeological record for soybean, encompassing the eastern Huanghe (Yellow River) basin in North China, South Korea, and Japan (Figure 2). If Zhao [11] is correct, domesticated soybean was present as early as the Longshan period (Figure 3) in North China suggesting that North China was, indeed, a region where soybean was domesticated. Archaeologists are collecting soybean measurements assuming that they are, in fact, able to distinguish wild from domesticated soybean. The extent to which seed size can clarify issues related to soybean domestication, however, has not been explicitly examined. This paper critically reviews archaeological soybean size and its usefulness to understanding the relationship between people and soybean in East Asia. Interdisciplinary inquiry is critical in the study of domestication [12], but while research on domestication of other major world crops all incorporate the archaeological record, archaeological research has contributed little to understanding soybean domestication. This paper is an attempt to begin rectifying this situation. We emphasize data over which we have control or that we collected. We address several key questions pertaining to soybean seed size. Do seed dimensions correlate with chronology? That is, is there evidence of seed size and shape selection over time? Are regional differences in soybean seed size apparent? Finally we evaluate whether the new data inform our understanding of soybean domestication.

## Materials and Methods

**Figure 4:**
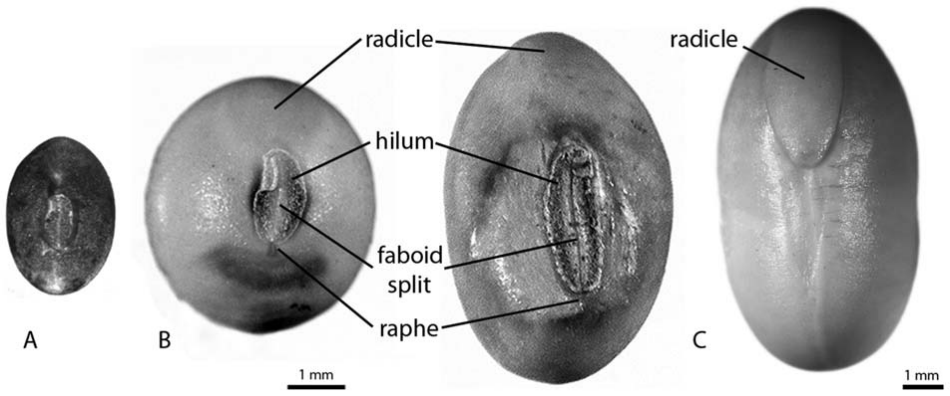
Hilar view of reference soybean. A. Modern wild soybean (Accession No. IT209387, dark colored seed, left). B. two variations of a small-seeded domesticated soybean (Accession No. IT209387, light colored seeds, right) illustrating main characteristics. The seed coat covers the radicle so only its outline is visible. C. Commercial soybean (separate scale) with seed coat removed to expose the radicle and imprint of the hilum.

Our sample consists of 949 charred archaeological soybean seeds and 180 charred and uncharred modern specimens, including a few examples of relevant published data. All the archaeological soybean seeds for this study were charred and relatively intact (Figure 4). Most were recovered using a flotation process based on the method used by Patty Jo Watson and her team in Kentucky and modified by Crawford to suit the circumstances of field research in Japan, then Korea, and finally China. Measurement of seed lengths and widths were obtained using a stereozoom microscope, and measured with an eyepiece reticule of 100 µm or measured with the assistance of NIS Elements or Adobe Photoshop (Table S1). For comparative purpose, three varieties of the genus *Glycine*, one domesticated (Accession No. IT209387) and two wild (IT822966 & 822967) were obtained from the Korean Agricultural Culture Collection (KACC) at the Rural Development Administration (RDA) of Korea. The cultivar is a traditional small-grained variety and was selected in order to provide a comparison with the relatively small seeds that dominate the archaeological collections. Modern wild soybean at the Huizui site in the Yiluo basin, China was collected by archaeologists of Institute of Archaeology, Chinese Academy of Social Sciences in November 2006, and the permission was given to the authors for further analysis.

**Figure 5:**
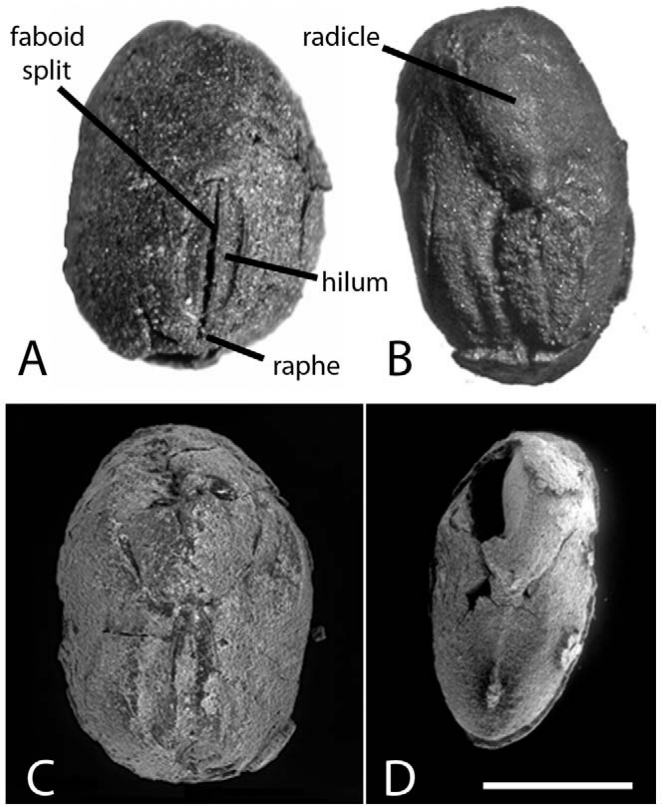
Hilar view of charred Neolithic soybean seeds. A. Dahecun. Specimen with seed coat attached showing a promiment hilum and faboid split; B. Jiahu. Most of the seed coat is absent exposing the bulbous radicle; C. SEM photograph of specimen from Dahecun with most of the seed coat missing but the hilum is still visible and the radicle is exposed; D. Pyeonggeodong. SEM photograph of soybean seed with patches of seed coat remaining; the bulbous form of the radicle is visible in the broken region at the top left of the image. Scale bar = 1 mm.

**Figure 6:**
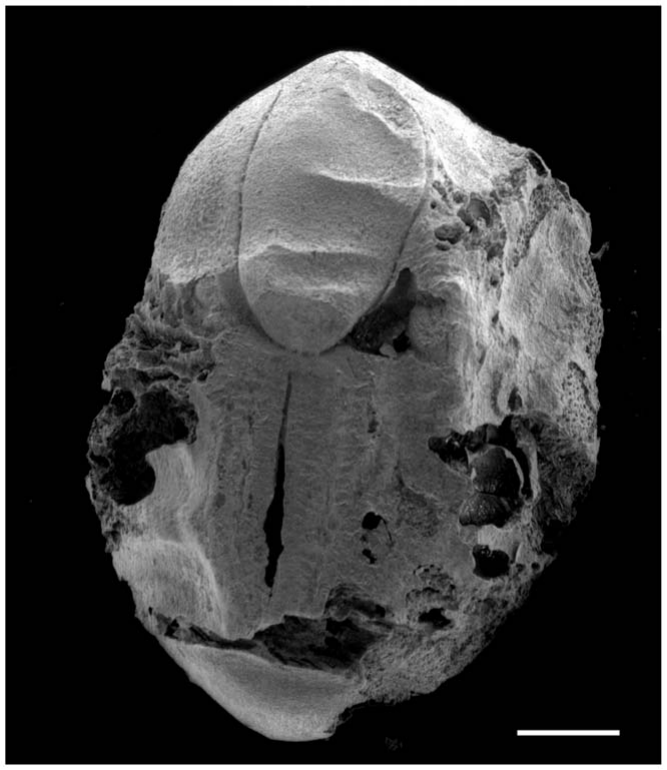
Hilar view of charred Mumun soybean. Daundong site. SEM photograph of seed with no seed coat; the radicle is at the top of the image while in the lower half is the faboid split in the hilar region. Scale bar = 1 mm.

**Figure 7:**
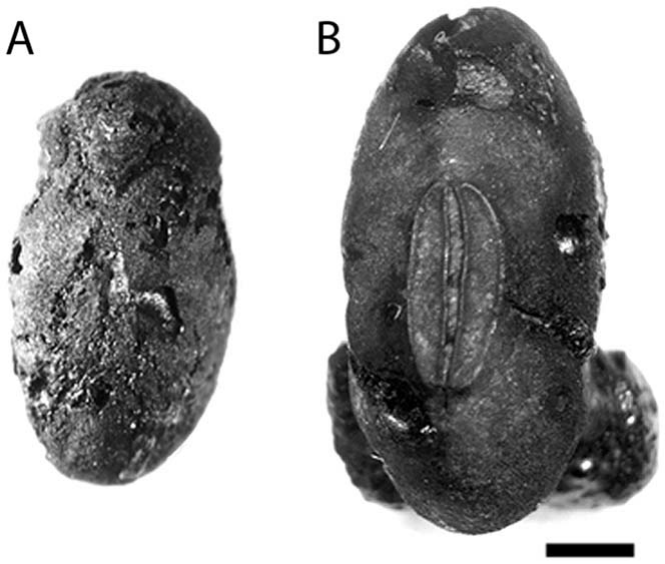
Hilar view of soybean seeds from Japan. A. Seed from the Late Jomon Usujiri Shogakko site; the entire seed coat is missing. B. Seed with well-preserved seed coat from the Middle Jomon occupation of the Shimoyakebe site. Scale bar = 1 mm.

**Figure 8:**
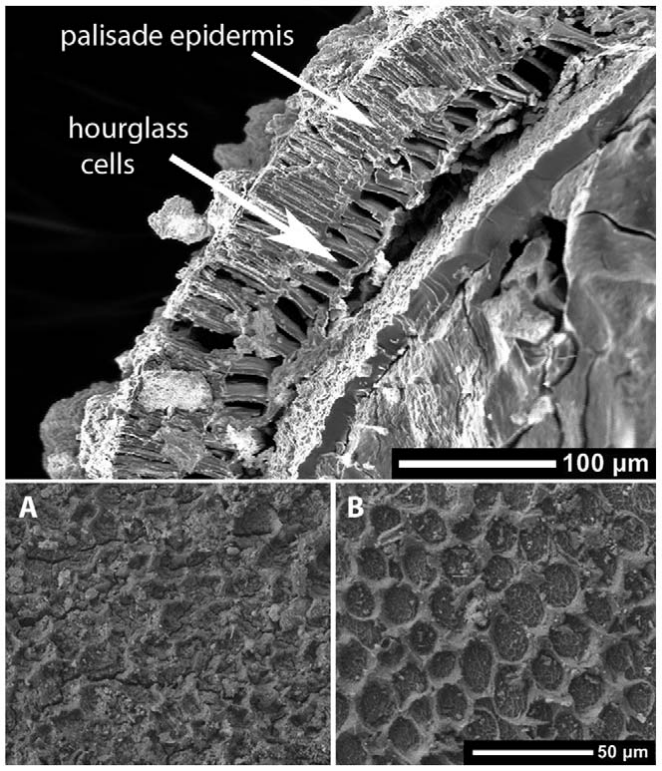
SEM photo illustrating details of a mature seed coat cross-section of a charred seed from Dahecun. Detail of seed coat surface on Dahecun seed (A) and modern wild seed (B) illustrating the typical honeycomb pattern of the bloom on seed coat exterior that shows that the seed had a dull luster. Such blooms are characteristic of both wild and domesticated soybean and variations of the bloom deposits determine whether a seed is shiny or dull.

The identification of the charred remains as soybean is based on comparisons with modern reference specimens. The seeds are clearly Fabaceae based on the bilateral symmetry, hilum, and radicle shape (Figures 5, 6, 7). The faboid groove places the seeds in the subfamily Faboideae [13]. The embryonic axis is straight, hilum curved, and the radicle is bulbose with a curved tip and less than half the length of the seed. The seed coats, when retained, have structurally supportive, hourglass cells and palisaded epidermis overlying a thin parenchyma layer [14] (terminology follows [13]), all traits of *Glycine* (Figure 8). The seeds that retain a seed coat appear to be mature because the seed coat function changes from being the main conduit of nutrients and metabolically active early in its development to mostly dead cells that protect the seed [14]. Whether all seeds in the studied samples are mature is uncertain but the seed coat structure suggests that they are.

**Figure 9:**
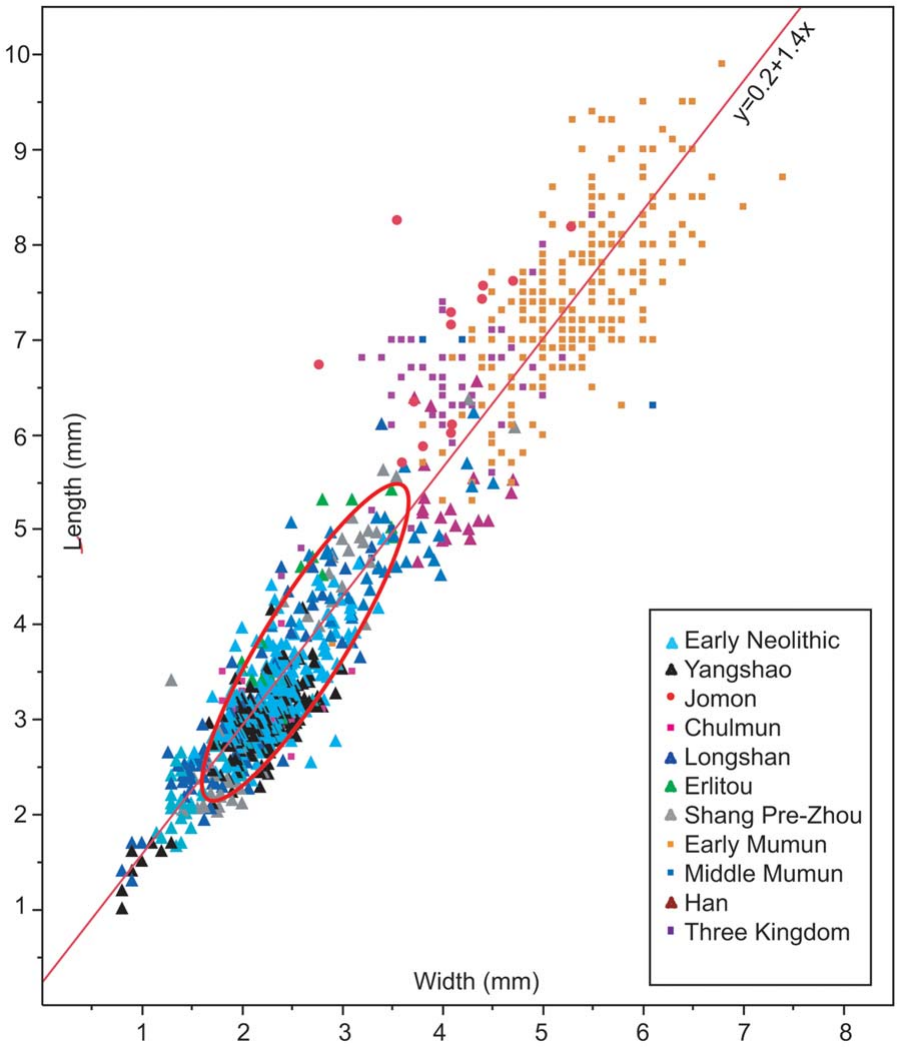
Scatter plot of length vs. width of archaeological soybean. The density ellipse (red) represents the 90 percent confidence limit of the regression line for modern wild soybean.

Domesticated seeds may be distinguished from their wild counterparts, particularly in the case of wheat, using computer-assisted morphometry whereas seed length and width measurements may not [15]. We do not employ computer-assisted morphometry in this study because we are evaluating the existing database of length (L) and width (W) measurements of archaeological soybean. Thickness (T) is not consistently represented in the available data so our discussion is somewhat limited to the available thickness measurements. The maximum dimension of the side where the radicle (embryonic root) and hilum are visible (embryonic axis) is mistakenly reported as thickness rather than width in most archaeological reports (Figure 4). We follow this convention rather than the botanical one in order to be consistent with other reports. We also consider the effectiveness of the ratios of L/W, LxW, and LxWxT to quantify seed shape and size. Although botanists do not consider seed shape to be a DRT, archeological reports occasionally refer to apparent differences in shape (L and W proportions in particular) (e.g. [16]) thus we consider the ratio of L to W. All length and width measurements contributed by the authors are plotted in Figure 9. Length and width have a strong, linear correlation (y = 0.2+1.4x) so we do not examine the variation of width in any detail.

## Results

### Soybean Taxonomy

The classification of domesticated plants and their wild ancestors commonly focuses on phenotypic differentiation and is rooted in genetics. Domesticated plants in general share varying degrees of dependence on people for their propagation and survival and have evolved characteristics (domestication related traits or DRT) related to this dependency. Domestication primarily makes the plant parts that are valued by people useful, or at least more useful than they are in the wild. The differences between domesticated plants and their wild relatives are not always clear and often impossible to discern in archaeological specimens. Some domesticates require more human investment than others and some are more genetically isolated and specialized, having undergone significant selection. Others are more similar to their wild relatives because selection has not been intensive. Furthermore, DRTs are also normally quantitative traits. In many domesticated plants, particularly the grasses (Poaceae) and nightshades (Solanaceae), DRTs are often linked in certain regions of the chromosome [17]. In soybean, these traits are found in only a few loci but they are not clustered [17]. In soybean, domestication likely created a genetic bottleneck that decreased genetic diversity, changed allele frequencies, increased allele linkage disequilibrium and eliminated rare alleles [3∶16666]. However, recent research demonstrates that alleles for such traits as determinancy (Table 1) that are rare in the wild are common in the crop [4].

**Table 1:** Principal Domestication Related Traits (DRT) Distinguishing Wild from Domesticated Soybean.

Wild	Domesticated	Source
Twining or procumbent annual vine	Mainly erect, annual, bushy with stout primary stem	[17], [18], [24]
Early flowering	Late flowering	[17], [18]
Small, black seeds	Small to large seeds with variable colors but mainly yellow	[17], [18], [24]
100-seed weight low	100-seed weight high	[24]
Indeterminate growth	Mainly determinate growth	[4]
Seed protein high, oil content low	Seed protein medium, oil content high	[24]
Pod dehisces naturally	Reduced dehiscence but trait is complex	[17]
Pod small to medium	Pod medium-large	[24]
Hard seededness (low permeability to water)	Variable hardness controlled by cuticle of seed coat	[14]

Domesticated plants are usually phenotypically distinctive and, because they are also cultivated, taxonomists often consider them to be separate species. In fact, niche difference is the main reason that Broich and Palmer [18] classify wild and domesticated soybean as distinct species: domesticated soybean being *Glycine max* and its wild relatives being *G. soja* (syn. *G. ussuriensis* or *G. formosana*). However, despite appearances and cultivation a domesticated plant and its wild relative can normally cross and produce a fertile F1 hybrid generation so they should be considered the same species [19]. Although soybean is self-pollinating, wild soybean and the crop can, and do, interbreed. Furthermore, the wild and domesticated forms have similar morphology, isozyme banding patterns and DNA polymorphisms [20]. The crop and wild ancestor are more appropriately classified as *G. max* subsp. *max* and *G. max* subsp. *soja* respectively [21], [22]. *G. max* subsp. *soja* is the only wild member of the subgenus *Soja* distributed in Korea, Japan, the Russian Far East, Taiwan, and most parts of China [7]. Throughout this paper we use the term “soybean” to refer to *G. max* without specifying the subspecies because of the ambiguity in the archaeological record. We do not wish to bias the terminology to favor either wild or domesticated soybean.

The characteristics that distinguish wild from domesticated soybean are quantitatively inherited [23]. These traits include pod and seed size (Table 1). Pod and seed size are closely related because the seeds fill the pod. The determinate growth of domesticated soybean plants means that vegetative production ceases because of the photosynthate demands of the developing seeds [4]. Determinancy is, therefore, related to increased fruit production and possibly the size of the seeds. Seed size could, therefore, be a proxy for the development of determinancy, a trait generally restricted to domesticated soybean but as far as we can tell, this has not been investigated. Some modern Asian landraces have wild-type traits such as small, impermeable seeds and twining habit likely resulting from crossing with wild soybean [17].

### Archaeological Background

This study focuses on the central and eastern Huanghe basin in China, southeastern Korea and to a limited degree Japan (Figure 1). Archaeobotanical research in Japan is addressing soybean but the database is not as comprehensive as it is in China and Korea. Research in the Yiluo valley along the central Huanghe where the first large urban centre (Erlitou) developed is particularly important for understanding agricultural development in North China because of its long sequence and extensive archaeological record of over 26 sites that have been sampled for plant remains [25], [26], [27].

Soybean is associated with agricultural systems that relied exclusively on dry crops as well as those that incorporated wet rice (*Oryza sativa*). Early Neolithic sites that have been systematically sampled include Xinglonggou (Xinglongwa culture) [28], Yuezhuang (Houli culture) [29] and Jiahu (Peiligang culture) [30] (Figure 5). No soybean is reported from the 7800–7600 cal BP contexts at Xinglonggou where broomcorn millet is common. Flotation samples from the Late Peiligang context at Bancun (ca. 7500 BP) recovered a single soybean seed [31]. Crawford has identified soybean in the Yuezhuang assemblage associated with broomcorn and foxtail millet as well as rice (ca. 8000 cal BP), but we do not know whether the rice was grown at Yuezhuang. An abundance of soybean seeds were recovered from all phases of occupation at Jiahu where rice was also an important resource [30], [32]. The earliest phase, based on radiocarbon dates on charcoal, thermoluminesence dates on ceramics and infrared-stimulated luminescence on sediments dates to 9000–8600 cal BP [33], [34], [35]. Elsewhere, soybean is associated with rice at the Late Neolithic Lianchengzhen site. How rice was produced at Liangchengzhen is under investigation but likely it was grown locally in wet fields. The earliest record of food production in the Yiluo region is associated with the Late Peiligang (7500 cal BP), the first Neolithic occupation there. Foxtail millet (*Setaria italica* subsp. *italica*) is part of the archaeological record at two Late Peiligang sites and was an important crop throughout the sequence with broomcorn millet becoming more common only in the Late Neolithic [25]. A wide range of annual weeds consistent with agricultural ecology is also a component of assemblages in the region, indicative of extensive anthropogenic open, sunlit areas to which wild soybean would have been attracted. However, the earliest soybean in the Yiluo region is from Late Yangshao Huizui and Zhaocheng sites. Dahecun (Figure S1), a Late Yangshao site less than 100 km from the Yiluo River, has a large quantity of soybean fortuitously recovered from a house floor (Figure 5). The seeds were mistakenly identified as sorghum (*Sorghum bicolour*) in the original report [36]. Soybean is more regularly recovered from the archaeological record beginning in the Late Yangshao through Shang mainly because sampling has been more extensive. For example, the Late Longshan Liangchengzhen site yielded soybean from various contexts, including pits, burials, pots, and other cultural activity areas although overall soybean density is low [37]. Intensive sampling of the Longshan Period, Jiaochangpu site, Shandong recovered nearly 10,000 soybean seeds [38].

The Korean soybean data are from multi-component sites in the Nam River valley (Oun 1, Okbang1/9, and Pyeonggeodong) as well as from the Daundong site in Ulsan (Figure 2). The Nam river sites are dated mainly to the Chulmun, Mumun, and early historic Three Kingdom (Figure 3). Dry crop production characterizes the Chulmun. Broomcorn and foxtail millet were the first crops in Korea, appearing as early as 5500 cal BP [6], [39]. Legumes appear to have become important shortly thereafter. Flotation samples from pits and house floors at Pyeonggeodong (Figures S2 and S3) in the Nam River valley recovered charred millets and two legumes, azuki (*Vigna* cf. *angularis*) and soybean (*Glycine max*). Soybean from Pyeonggeodong (Figure 5) is dated to 4840–4650 cal BP (Figure 1). Chulmun people exploited diverse wild plants including nuts (Genera *Quercus*, *Juglans*), fruits (*Actinidia*, *Prunus*, *Vitis*, *Cornus*), small-seeded annuals (*Chenopodium*, *Polygonum*, *Panicum*, *Setaria*) and possibly other herbs, totalling over 20 species [39]. This Chulmun procurement system does not appear to represent as intensive cultivation as in China at the same time but anthropogenic impacts on open habitats and millet cultivation from 5500 cal BP probably facilitated legumes thriving and potentially being collected and cultivated.

Intensive agriculture was established by the beginning of the Mumun period around 3500 BP. Rice, wheat (*Triticum* cf. *aestivum*), barley (*Hordeum vulgare*), azuki, and soybean were cultivated by then [6]. Irrigated rice paddy fields are well documented too [39]. Soybean densities in dwelling sites are high. For example, floor fills of a pit-house at the Daundong site contained thousands of charred soybean at a density of 307 per liter of sediment (Figure 6). Soybean is also found in a variety of Mumun contexts along the Nam River as well. Soybean remained a significant crop in the subsequent Three Kingdom period [39].

Charred archaeological soybean specimens from Japan date from the Middle to Late Jomon periods (Figure 7). Recent analysis of grain impression on pottery, however, indicates the presence of *Glycine* in the archaeological record as early as the Initial Jomon. The grain impression at the Yamanokami site in Chubu measures 5.62 by 3.86 mm and is classified as wild soybean [40]. *Glycine* impressions are also reported from the Early Jomon in the Kanto and Chubu districts [41]. Soybean seeds from Yayoi and later sites in Japan are not considered here. The largest charred soybean seed sample is from a Middle Jomon midden, largely composed of walnut shell (*Juglans mandshurica*), at Shimoyakebe, Tokyo (Figure S4) [42]. The AMS date on *Glycine* (PLD-9088) is consistent with dates on walnut shell and another legume (MTC05837) (Figure 1). These seeds, originally classified as a type of *Vigna* (Type B) in the site report [43], are actually soybean [16], [44]. A charred Late Jomon specimen (Figure 7) is from the Usujiri Shogakko site in southwestern Hokkaido (Figure S5). The Jomon in these locations did not have a Chinese or Korean form of agriculture although people were likely the keystone species, at least in northeastern Japan during these periods and were involved in extensive anthropogenic processes that included plant management [45]. As in China and Korea, habitats in which wild soybean could flourish during the Jomon were common.

### Comparisons To Modern Glycine

Complicating the analysis is the possible effect of charring on soybean seeds. Some researchers estimate that soybean lengths and widths reduce in size by 15% after charring [46]. Other carbonization experiments, however, indicate that the duration and temperature of firing as well as the heating rate differentially affect the size of charred seeds of pea [47], [48] and sunflower [48]. Soybean was likely affected differentially by these conditions as well as water content of the seeds, so a simple formula that converts sizes from charred to fresh dimensions without knowing the specific charring conditions is not presently feasible.

**Figure 10:**
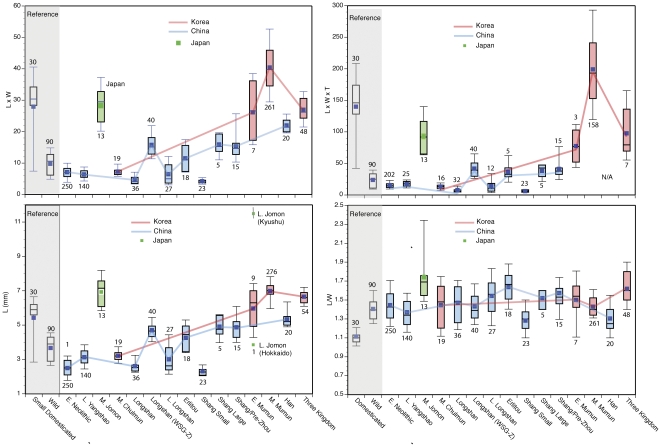
Box plots of soybean seed length, length by width, length **×** width, and length **×** width **×** thickness. Numbers indicate seed numbers measured. Measurements from different sites for each period are combined. The top, bottom and line through the middle of the box correspond to the 75th percentile (top quartile), 25th percentile (bottom quartile) and 50th percentile (median) respectively. The whiskers on the bottom extend from the 10th percentile (bottom decile) and top 90^th^ percentile (top decile). Solid boxes represent means. Samples represented by a box have one or only a few specimens. Only the range is published for the Late Jomon sample from Kyushu.

In order to facilitate a comparison with the archaeological seed measurements, we acquired 30 modern domesticated soybean seeds (IT209387) and 60 wild soybean seed (IT282966, IT 282967) from the KACC of the RDA. We also collected wild soybean at the Huizui site in November 2006 by cutting stalks with pods that were almost mature but not yet open. As illustrated in Figure 10, the lower 23 percentiles of the length values of modern domesticated variety overlap the values for wild ones from both Korea and China.

### China

The 258 beans from Jiahu, including the published data [30] and our own measurements, average 3.1 by 2.2 mm (length by width) and are among the smallest soybean seeds in this study (Figure 10). Two seeds from Yuezhuang are also quite small, averaging 2.6 by 1.8 mm.

The size ranges of soybean seeds from Longshan sites are quite variable. For example, specimens from Wangchenggang in Henan and Zhouyuan in Shaanxi (listed as Longshan-WSG-Z in Figure 10) have significantly larger specimens than those from other Longshan sites (Shantaisi and Xijinghceng in Henan) and those from Late Longshan sites (listed as L Longshan and include the Yiluo basin Huizui and Jianxicun sites and Liangchengzhen in Shandong). Soybean seeds from Jiaochangpu average 3.4 by 2.6 mm [38] (not included in our charts because only the averages are published) and are similar in size to the latter group. Zhouyuan and Wangchenggang are multi-component sites, and soybean was also recovered from the later periods in both sites (Proto-Zhou and Erligang). AMS dates on soybean have not been obtained from these sites. Soybean at the two sites may be evidence of the evolution of a large seeded soybean variety/landrace during the Longshan period. Without AMS-dated soybean seeds the hypothesis cannot be tested yet.

Two Erlitou sites (Huizui and Xinzhai in Henan), three Shang sites (Tianposhuiku N and Wangchenggang in Henan, and Daxinzhuan in Shandong), and one Proto-Zhou site (Zhouyuan in Shaanxi) yielded 46 measurable soybean seeds. The soybean population at Zaojiaoshu, another Erlitou site in the Yiluo basin [49], for which only a summary of soybean measurements is available, is similar in size to the other Erlitou soybean populations. Finally, 28 soybean seeds from the Shang period Daxinzhuang site consist of distinct small and large types: large specimens (listed as Shang-large in Figure 6) resemble soybean from the other Shang and Proto-Zhou (listed as Shang/Proto-Zhou), while the small seeds are among the smallest recovered from China to date.

### Korea

Soybean seeds at the Chulmun Pyeonggeodong site are within the size range of the Early and Middle Neolithic China specimens as well as the Late Longshan soybean, a roughly contemporaneous population (Figure 10). They are about half the size of soybean seeds that are within the size range of domesticated soybean at Mumun sites in the Nam River valley (Figure 6). Both the Chulmun and Late Longshan soybean are significantly smaller than the Mumun population. Mumun soybean lengths range from 3.8 to 9.9 mm. Most of the Mumun specimens are longer and wider than any earlier archaeological soybean in our samples except for those from Japan (Figure 10). Even the contemporaneous Shang and Proto-Zhou population in China mostly fall below the size range of Early Mumun. L/W ratios of Mumun soybean (1.0–2.1) are also larger than other archaeological specimens except for the Three-kingdom specimens. The morphology of these specimens resembles smaller ovate domesticated varieties [6].

### Japan

DNA analysis of charred legumes, not directly dated, from the Early Jomon (ca. 5800–5300 cal BP) component of the Sannai Maruyama site indicates that some may be *Glycine* sp. [43]. The identification of the seeds as *Glycine*, however, has been questioned because of their morphology [40]. Nevertheless, the Middle Jomon Shimoyakebe specimens are the largest specimens in East Asia at the time (ca. 5000 cal. BP) (Figures 7, 10). Soybean impressions have also been identified on pottery at the late Early Jomon Tenjin [50] and the Middle Jomon Sakenomiba and Meotoshi sites [16]. Six soybean impressions on pottery from the Late Jomon in Kyushu [16] are estimated to be 10.2–11.8 mm by 6.5–7.9 mm wide, the largest soybean seeds in this study. The Jomon soybean archaeological record is poorly known because flotation sampling is only regionally common. Furthermore the 100 Jomon legume reports (usually *Vigna* sp.) need to be reviewed. Ongoing research indicates that soybean may, indeed, be more extensively represented in the Jomon archaeological record. For example Crawford reexamined an unknown seed from the Late Jomon Usujiri Shogakko site (ca. 3500 cal BP). The seed is soybean (4.5 by 2.3 by 3.1 mm). Soybean has not been reported in the Hokkaido Jomon record previously [51].

## Discussion

Soybean seed size tends to increase over time in each region (Figure 9) although the chronological pattern of size change differs among the three regions (Figure 10). Small seeds, often smaller than wild seeds, are common at Chinese Neolithic sites (Houli, Peiligang, Yangashao, and Longshan periods). Because seed size also depends on harvest timing, the extremely small seeds in some of these assemblages may be undeveloped or immature seeds or extremely late season seeds. Most of the immature seed weight is in the soft and highly metabolic seed coat [14]. The specimens in our sample (Figure 3) appear to be well developed with missing or mature seed coats so none of our examples are particularly immature. Domesticated soybean seeds gain weight and size and become more spherical for the first four weeks after blooming, then they lose weight and reduce in size as they continue to mature and by seven weeks after blooming are significantly smaller [52]. Much of the weight loss in maturity is due to desiccation. Charring of undeveloped or immature seeds may also reduce the seed size because of their high moisture content. The reason for the extremely small seeds in the Chinese record is, as yet, unclear. With the exception of the smallest charred seeds and the Longshan period Zhouyuan and Wangchenggang specimens (listed as Longshan-WCG-Z in Figure 10), the Chinese Neolithic soybean seeds are in the wild size range. Seed lengths overlap with the lower 25th percentile of the small, reference domesticated soybean and most wild soybean. LxW products also have some overlap with the smaller domesticated seeds but LxWxT products are almost entirely in the wild range (Figure 10) indicating little evidence for seed size selection during the Chinese Neolithic. However, by the Erlitou-Shang period the length and LxW and LxWxT products increase significantly and bimodality is evident in the Shang sample at Daxinzhuang (identified as “Shang Small” in Figure 10). This indicates that two types of soybean, probably wild/weedy and domesticated soybean, are present. In Korea, seed sizes increases significantly by the Early Mumun (Figure 10). The increase in size occurred during the transition from Chulmun to Mumun (4500–3500 BP) but we have no samples from that period. The Japanese data contrast markedly with both the Korean and Chinese data. By Middle Jomon the seeds are significantly larger than any wild soybean seeds or contemporaneous soybean seeds in Korea and China (Figure 10). They are similar in size to Mumun seeds dating over 2000 years later. The trend to increased seed size continues through 3600 cal BP (Figure 10) when Late Jomon soybeans from Kyushu appear to be in the size range of some large, modern varieties.

The Three Kingdom sample in Korea suggests that at least two varieties of soybean are present from the Middle Mumun onwards. The smaller sizes of these historic period samples compared to the Mumun specimens may be an artifact of sample size as five-times more seeds were measured from the Mumun context. The larger sizes and high concentrations of soybean seeds in a house and a field indicate that the Middle Mumun Daundong and Three-Kingdom soybean are domesticated. The Chinese soybean seeds continue to increase in size through the Han period (2170–1730 BP) (Figure 10) but they are still smaller than the older Jomon, Mumun, and Three Kingdom period seeds. Selection of larger seeded varieties appears to have occurred much earlier outside China, either in Korea and Japan.

**Figure 11:**
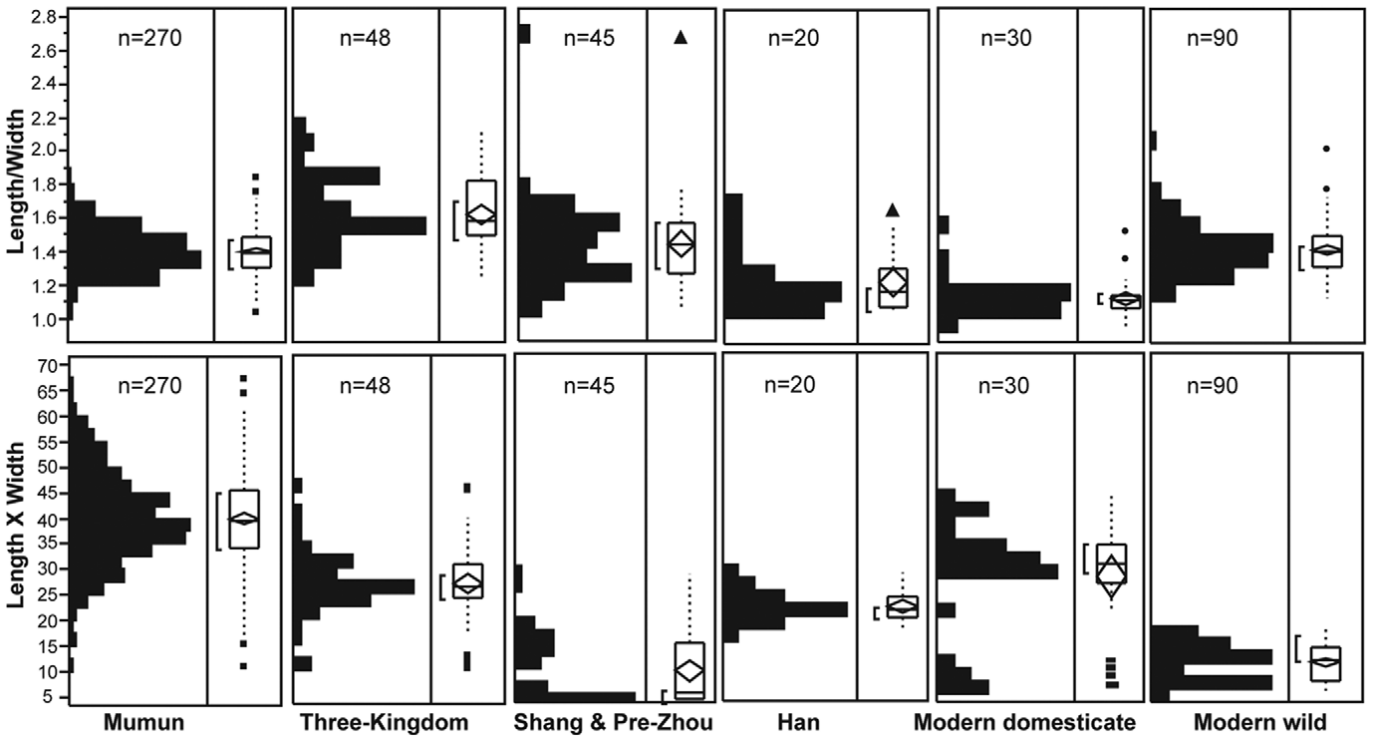
Comparison of shape and size of late soybean populations.

Despite shape not being considered a DRT, the L/W ratios of the modern wild and domesticated soybean reference specimens do not overlap significantly. The wild soybean seeds have higher ratios because they are elongate (rectangular) rather than square (Figure 10). The archaeological seeds tend to be relatively elongate. The large Middle Jomon seeds are especially long in relation to their width. Otherwise the archaeological populations have a greater range of shape variation than either of the reference taxa. Late Yangshao, Late Chulmun, Longshan, Early Mumun, small Shang, and Han populations all have seeds that overlap with the upper 25 percent whisker in the box plot of the domesticated reference material L/W ratio (Figures 10, 11). At this stage of our research shape (L/W ratio) does not appear to trend toward the less elongate shape. Harvest time differences may be an important factor in the shape variations of the archaeological specimens.

The modern domesticated soybean seeds measured for this study represent a traditional Korean landrace with the smallest seeds we could obtain, so we hypothesized that it would be similar in size and shape to at least some of the archaeological populations. The differences, however, are greater than anticipated. The larger archaeological soybean samples have a seed shape distinct from that of the small-seeded, modern Korean domesticate. Furthermore, the small number of seeds in our domesticated soybean reference sample includes both spheroidal and narrow forms in hilar view (Figure 4). The seed size distribution of this sample is also bimodal. Future research should include modern domesticated landraces with various forms and sizes in order to further understand variation in the archaeological populations.

We hypothesize that seed size selection occurred in all three regions and the significant size increases in Japan, Korea, and China indicate that domestication was well under way before the Middle Jomon in Japan, between the Late Chulmun and Early Mumun in Korea and by the Shang period in China. Given that LWT product is the only statistic that distinguishes the wild and domesticated reference material and that the LWT product trend is similar to the LW product and L, archaeologists should routinely measure soybean thickness. L/W ratios also distinguish wild and domesticated reference material, but the pattern for the archaeological material does not seem to have much chronological information.

How does soybean seed size inform us about the domestication of this crop? The ambiguity of the seed size data in determining the earliest stages of the domestication process, particularly in China, is not surprising. In soybean, seed size increase appears to be the result of an accumulation of minor changes at quantitative trait loci (QTL) [4]. The size increase may have an as yet to be determined link to determinate growth, a trait rare in the wild but common in domesticated soybean. In other Old World Fabaceae such as peas and lentils, seed size increase is gradual with considerable overlap between wild and domesticated varieties [53], [54]. Seed size similarly may not indicate when soybean was initially being domesticated and we suggest that it was being cultivated before significant seed size changes are apparent, consistent with the domestication process in other legumes [53]. Selection of DRTs may have initially focussed on pod dehiscence and seed hardness (water permeability), traits not yet discernable in the archaeological record. Tian et al. [4] interpret their DNA analysis to mean that growth habit (erect, bushy plants with determinate growth) selection occurred before the radiation of all the lineages of Chinese soybean landraces just after or during the major domestication transition. Distinguishing these traits in charred specimens without aDNA is not presently feasible.

The large deposits of seeds found in Yangshao, Longshan, Chulmun, and Jomon sites indicate a long history of soybean and human interaction. Soybean cultivation and selection for larger seeds occurred by 5000 BP in Japan and at least 3500 BP in Korea and China. Certainly the setting for selection of DRTs is apparent 9000–5000 BP in China, Korea, and Japan.

Historic evidence suggesting that soybean cultivation revolutionized agriculture in the Eastern Zhou Dynasty of China shortly after its introduction around 2510 BP from the northeast [55] should not be taken to mean that soybean was domesticated in only one location and that it was the Northeast. The earliest appearance of soybean in NE China is at the Xinglonggou site (Locality 3) where it appears shortly after 4000 cal BP [28]. The specimens at Xinglonggou are called “domesticated” [28] but the criteria for this identification are not clear. Several finds of soybean in Jilin and Heilongjiang provinces indicate that soybean spread throughout the northeast after 3000 BP during the Zhou period [56]. This record, sparse though it is, suggests that soybean was introduced to, rather than from, NE China. Botanical data are consistent with this view too. The highest diversity among modern soybean landraces is found in the Huanghe region so, following Vavilov's idea of crop origins [57], Dong *et al.* [58] and Li *et al.* [59] suggest that soybean was domesticated in one location, the Huanghe valley, rather than somewhere in NE China or elsewhere in East Asia. Seed composition and seed protein electrophoresis [60] have also been used to support this view. Tian *et al.* [4] also favor the Huanghe hypothesis but they only examined Chinese landraces. Furthermore, diversity only indicates that a crop has a long history in a region not that it was domesticated there. However, the early record for soybean and evidence for selection of larger seeded soybean in the Huanghe valley, and the distinct populations in China from 9000 to 3500 cal BP compared to those from Korea and Japan, is consistent with independent domestication there.

Others, based on historical documents, suggest that soybean was domesticated in South China or in multiple centers in and outside China [61], [62]. Archaeological evidence for soybean is general absent prior to the Han period (2170–1730 cal BP) in southern China. We are aware of only one report from the Bashidang site (8000 cal BP) [63], but the identification criteria are not outlined [63]. No early written records mention soybean in South China. Sampling is likely not responsible for the general absence of archaeological soybean there. Many waterlogged sites dating to 8000–4000 cal BP in the Yangzi basin (e.g. Kuahuqiao, Hemudu, Luojiajiao, and Chengtoushan) have large quantities of well preserved plant remains. None have yielded confirmed soybean remains. At the moment, we have no archaeological evidence that a domestication of soybean occurred in southern China.

The hypothesis of multiple domestications in East Asia comes from broad comparative studies of phylogenetic data [62], [64], [65], [66]. For example, the highly differentiated gene pools of Chinese and Japanese soybean point to their separate origins [64], [66]. Furthermore, distinctions in the chloroplast genomes cannot be explained by pollen flow from wild to domesticated soybean but require that wild plants were taken into cultivation more than once [64], [66]. The archaeological data are consistent with the multiple origins hypothesis. The archaeological record for soybean in Japan raises a question about a complex issue involving Zhou Dynasty texts [55]. These texts describe soybean as having been introduced from the northeast. This, combined with the absence of a record for soybean in the Huanghe valley at the time, suggested that northeast China was the source of domesticated soybean [67]. Now that we have an extensive record for Neolithic soybean in the Huanghe valley, as well as from Japan and Korea, another interpretation may be worth testing. Could Japan have been a source of a large-seeded landrace of domesticated soybean that spread to Korea and subsequently to China? This record, if it is to be trusted, could be referring to a particular landrace (rather than soybean in general) that would have been quite different from the landraces already grown in China by 2500 BP. We admit that this is highly speculative and difficult to test at this stage of soybean research. Larger Erlitou-Shang soybean remains seem to match the record in *Shijing* and *Xiaxiaozheng* indicating a tradition of soybean cultivation in China prior to 3000 BP [68]. Furthermore, soybean-bearing sites are concentrated in the central and eastern Huanghe basin in China where a long history of intensive agriculture and high population density are well documented [26]. Finally, the recent genome sequence comparison of wild and domesticated soybean that points to a complex domestication history for soybean [24], [69] is supported by our analysis.

## Supporting Information

Figure S1 **Yangshao Houses at the Dahecun site, Henan, China.** Remains of houses at Dahecun, from left to right: F2, F1, F3. The pottery jar containing soybeans was discovered on the northeast corner of F2, indicated with a red dot. The jar however is now misplaced in the central area near the eastern wall (photo taken by Li Liu at the Dahecun Museum, Zhengzhou, 2010).
(TIF)

Figure S2 **Aerial photograph of the Chulmun Pyeonggeodong site, Korea.** Pyeonggeodong (Figure 2) is a multi-component site, situated in alluvial flats along the Nam River, southeastern Korea (highlighted in the inset). The area discussed is within the green boundary. Numerous structures, including pit houses, dolmen burials, farming fields, and hunting traps were recovered in an extensive area of 15 ha, dating from 5000 to 1200 BP. Analysis of plant remains from these features is ongoing. Most soybean specimens were found in Chulmun pits, including the one in Figure S3.
(TIF)

Figure S3 **Chulmun pit at the Pyeonggeodong site.** The pit feature (no. 28) contained charred soybean seeds along with azuki beans that are AMS-dated to 4830–4650 cal. BP (UCI60749, Table 1).
(TIF)

Figure S4 **The Shimoyakebe site, Japan.** A. Overview of the waterlogged, Middle Jomon walnut midden with well preserved wood; B. detail of walnut midden from where soybean sample was recovered.
(TIF)

Figure S5 **Pit House at Late Jomon Usujiri Shogakko site, Japan.** Several of the pit houses excavated in 1977 at Ususjiri Shogakko were destroyed by fire. Parts of the charred superstructure lie on the floor. Flotation samples were collected from the floor among the charred wood fragments.
(TIF)

Table S1 **Soybean seed sizes from the research area.**
(DOCX)

## References

[1] Graham PH, Vance CP (2003). Legumes: importance and constraints to greater use.. Plant Physiol.

[2] Ross-Ibarra J, Morrell PL, Gaut BS (2007). Plant domestication, a unique opportunity to identify the genetic basis of adaptation.. Proc Natl Acad Sci USA.

[3] Hyten DL, Song Q, Zhu Y, Choi IY, Nelson RL (2006). Impacts of genetic bottlenecks on soybean genome diversity.. Proc Natl Acad Sci USA.

[4] Tian Z, Wang X, Lee R, Li Y, Specht JE (2010). Artificial selection for determinate growth habit in soybean.. Proc Natl Acad Sci USA.

[5] Carter TEI, Nelson RL, Sneller CH, Cui Z, Boerma HR, Specht JE (2004). Genetic diversity in soybean.. Soybeans: improvement, production, and uses.

[6] Crawford GW, Lee G-A (2003). Agricultural origins in Korea.. Antiquity.

[7] Hymowitz T, Boerma HR, Specht JE (2004). Speciation and cytogenetics.. Soybeans: Improvement, production, and uses.

[8] Reimer PJ, Baillie MGL, Bard E, Bayliss A, Beck JW (2009). Intcal09 and marine09 radiocarbon age calibration curves, 0–50,000 years cal BP.. Radiocarbon.

[9] Stuiver M, Reimer PJ (1993). Extended 14C database and revised CALIB radiocarbon calibration program.. Radiocarbon.

[10] Stuiver M, Reimer PJ, Reimer RW (2005).

[11] Zhao Z, Zhao Z (2010). Flotation results from the Wangchenggang site, Dengfeng County of Henan.. Palaeoethnobotany: theories, method and practices.

[12] Zeder MA, Bradley DG, Emshwiller E, Smith BD, Zeder MA, Bradley DG, Emshwiller E, Smith BD (2006). Documenting domestication: bringing together plants, animals, archaeology, and genetics.. Documenting domestication: new genetic and archaeological paradigms.

[13] Kirkbride JH, Gunn CR, Weitman AL (2003). Fruits and seeds of genera in the subfamily Faboideae (Fabaceae).

[14] Qutob D, Ma F, Peterson CA, Bernards MA, Gijzen M (2008). Structural and permeability properties of the soybean seed coat.. Botany.

[15] Rovner I, Gyulia F (2007). Computer-assisted morphometry: a new method for assessing and distinguishing morphological variation in wild and domestic seed populations.. Econ Bot.

[16] Obata H, Sasaki Y, Senba Y (2007). Impressions on pottery revealed cultivation of *Glycine max* subsp. *max* (soybean) in the late to latest Jomon preriods in Kyushu Island.. Jap J Hist Bot.

[17] Liu B, Fujita T, Yan ZH, Sakamoto S, Xu D (2007). QTL mapping of domestication-related traits in soybean (*Glycine max*).. Ann Bot.

[18] Broich SL, Palmer RG (1981). Evolutionary studies of the soybean: The frequency and distribution of alleles among collections of *Glycine max* and *G. soja* of various origin.. Euphytica.

[19] Harlan JR (1992). Crops and man.

[20] Hancock JF (2004). Plant evolution and the origin of crop species.

[21] USDA ARS National Genetic Resources Program (2011). Taxon: *Glycine soja* Siebold & Zucc.: Germplasm Resources Information Network - (GRIN) [Online Database]..

[22] USDA ARS National Genetic Resources Program (2011). Taxon: *Glycine max* (L.) Merr.: Germplasm Resources Information Network - (GRIN) [Online Database]..

[23] Schmutz J, Cannon SB, Schlueter J, Ma J, Mitros T (2010). Genome sequence of the palaeopolyploid soybean.. Nature.

[24] Lam H-M, Xu X, Liu X, Chen W, Yang G (2010). Resequencing of 31 wild and cultivated soybean genomes identifies patterns of genetic diversity and selection.. Nat Genet.

[25] Lee G-A, Crawford GW, Liu L, Chen X (2007). Plants and people from the early Neolithic to Shang periods in North China.. Proc Natl Acad Sci USA.

[26] Liu L, Chen X, Lee YK, Wright H, Rosen A (2002–2004). Settlement patterns and development of soical complexity in the Yiluo region, north China.. J Field Archaeol.

[27] Rosen AM (2007). The role of environmental change in the development of complex societies in China: a study from the Huizui site.. B Indo-Pacific Prehist Assoc.

[28] Zhao Z (2005). Discussion of the Xinglonggou site flotation results and the origin of dry farming in northern China.. Antiquities of Eastern Asia.

[29] Crawford GW, Chen X, Wang J (2006). Houli culture rice from the Yuezhuang site, Jinan (in Chinese).. Dongfang Kaogu.

[30] Zhao Z, Zhao Z (2010). Flotation results from the Jiahu site, Wuyang county of Henan.. Palaeoethnobotany: theories, method and practices.

[31] Kong Z, Liu C, He D (1999). Plant remains unearthed from Zhuanglixi site in Tengzhou of Shangdong and their significance in environmental archaeology.. Kaogu.

[32] Liu L, Lee G-A, Zhang J (2007). Evidence for the beginning of rice domestication in China: a response to Fuller et al.. Holocene.

[33] Yang X-Y, Kadereit A, Wagner GA, Wagner I, Zhang J-Z (2005). TL and IRSL dating of Jiahu relics and sediments: clue of 7th millennium BC civilization in central China.. J Archaeol Sci.

[34] Zhang J, Harbottle G, Wang C, Kong Z (1999). Oldest playable musical instruments fround at Jiahu early Neolithic site in China.. Nature.

[35] Crawford GW, Shen C (1998). The origins of rice agriculture: recent progress in East Asia.. Antiquity.

[36] Liu L, Crawford GW, Lee G-A, Chen X, Ma X (2012). Re-analysis of the Yangshao “sorghum” remains at Dahecun in Zhengzhou.. Kaogu.

[37] Crawford GW, Underhill A, Zhao Z, Lee G-A, Feinman G (2005). Late Neolithic plant remains from northern China: preliminary results from Liangchengzhen, Shangdong.. Curr Anthropol.

[38] Zhao Z (2004). Comparison and analysis of the characteristics of agricultural production at Liangchengzhen and Jiaochangpu during the Longshan period.. Dongfang Kaogu.

[39] Lee G-A (2003). Changes in subsistence systems in southern Korea from the Chulmun to Mumun periods: arachaeobotanical investigation [dissertation].

[40] Obata H (2011). Jomon agriculture and paleoethnobotany in Northeast Asia.

[41] Obata H, Manabe A, Kenkyukai KJ (2011). Issues on the early agriculture in Korea and Japan, based on recent archaeobotanical studies (in Japanese and Korean).. Current research on the Neolithic period in Japan and Korea: proceedings of the 9th conference of the Kyushu Jomon Kenkyukai and the Korean Neolithic Research Society.

[42] Kudo Y, Sasaki Y (2010). Characterization of plant remains on Jomon pottery excavated from the Shimoyakebe site, Tokyo, Japan.. B Natl Museum Jap Hist.

[43] Sakamoto S, Ishikawa R, Nakamura I, Sato Y, Shimamoto Y (2006). Species identification of 6,000-year-old beans from Sannai-Maruyama site, Aomori, Japan.. Jap Fossil Res.

[44] Sasaki Y, Kudo Y, Momohara A (2007). Utilization of plant resources reconstructed from plant macrofossils during the latter half of the Jomon period at the Shimo-yakebe site, Tokyo.. Jap Assoc Hist Bot.

[45] Crawford GW (2008). The Jomon in early agriculture discourse: issues arising from Matsui, Kanehara, and Pearson.. World Archaeol.

[46] Zhao Z (2007). Research on agriculture in the Central Plains from 2500 to 1500 BC.. Kagi Kaogu.

[47] Braadbaart F, Bergen P (2004). Digital imaging analysis of size and shape of wheat and pea upon heating under anoxic conditions as a function of the temperature.. Veget Hist Archaeobot.

[48] Braadbarrt F, Wright PJ (2007). Changes in mass and dimensions of sunflower (Helianthus annuus L.).. Econ Bot.

[49] Luoyang Relics Team, editor (2002). Luoyang zaojiaoshu.

[50] Nakayama S, Nagasawa H, Hosaka Y, Noshiro Y (2009). The Tenjin site.. B Yamanashi Prefectural Museum.

[51] Crawford GW (1983). Paleoethnobotany of the Kameda Peninsula Jomon.

[52] Carlson JB, Lerston NR, Boerma HR, Specht JE (2004). Reproductive morphology.. Soybeans: improvement, production,and uses. 3rd ed.

[53] Fuller DQ (2007). Contrasting patterns in crop domestication and domestication rates: recent archaeobotanical insights from the Old World.. Ann Bot.

[54] Zohary DaMH (1973). Domestication of pulses in the Old World.. Science.

[55] Ho P-T (1975). The cradle of the east: an inquiry into the indigenous origins of techniques and ideas of Neolithic and early historic China, 5000–1000 B.C.

[56] Liu L, Shu S, Li F (1987). Preliminary identification of carbonised soybeans unearthed from Yongji, Jilin.. Kaogu.

[57] Vavilov NI, Love D, translator (2009). Origin and geography of cultivated plants..

[58] Dong YS, Zhao LM, Liu B, Wang ZW, Jin ZQ (2004). The genetic diversity of cultivated soybean grown in China.. Theor Appl Genet.

[59] Li YH, Li W, Zhang C, Yang L, Chang RZ (2010). Genetic diversity in domesticated soybean (*Glycine max*) and its wild progenitor (*Glycine soja*) for simple sequence repeat and single-nucleotide polymorphism loci.. New Phytol.

[60] Xu B, Zheng H, Lu Q, Zhao S, Zou S (1986). Three new evidence of the original area of soybean.. Soybean Science.

[61] Choi DH (2004). The origins of cultivated soybean and Korean Peninsula (in Chinese).. Nongye Kaogu.

[62] Zhao T, Gai J (2004). The origin and evolution of cultivated soybean [*Glycine max* (L.) Merr.]).. Sci Agr Sinica.

[63] Hunan Institute of Archaeology (2006). Pengtoushan and Bashidang.

[64] Abe J, Xu D, Suzuki Y, Kanazawa A (2003). Soybean germplasm pools in Asia revealed by nuclear SSRs.. Theor Appl Genet.

[65] Gai J, Xu D, Gao Z, Shimamoto Y, Abe J (2000). Studies on the evolutionary relationship among eco-types of *G. max* and *G. soja* in China.. Acta Agron Sinica.

[66] Xu D, Abe J, Gai J, Shimamoto Y (2002). Diversity of chloroplast DNA SSRs in wild and cultivated soybeans: evidence for multiple origins of cultivated soybean.. Theor Appl Genet.

[67] Shelach G (2000). The Earliest Neolithic Cultures of Northeast China; Recent Discoveries and New Perspectives on the Beginning of Agriculture.. J World Prehist.

[68] Han Z (1995). Shijing Yizhu.

[69] Kim MY, Lee S, Van K, Kim T-H, Jeong S-C (2010). Whole-genome sequencing and intensive analysis of the undomesticated soybean (*Glycine soja* Sieb. and Zucc.) genome.. Proc Natl Acad Sci USA.

