# Supplementary figures and images for: Archaeological Soybean (Glycine max) in East Asia: Does Size Matter?
Source: PLoS One. 2011 Nov 4;6(11):e26720. doi: 10.1371/journal.pone.0026720 (PMC3208558; doi:10.1371/journal.pone.0026720)

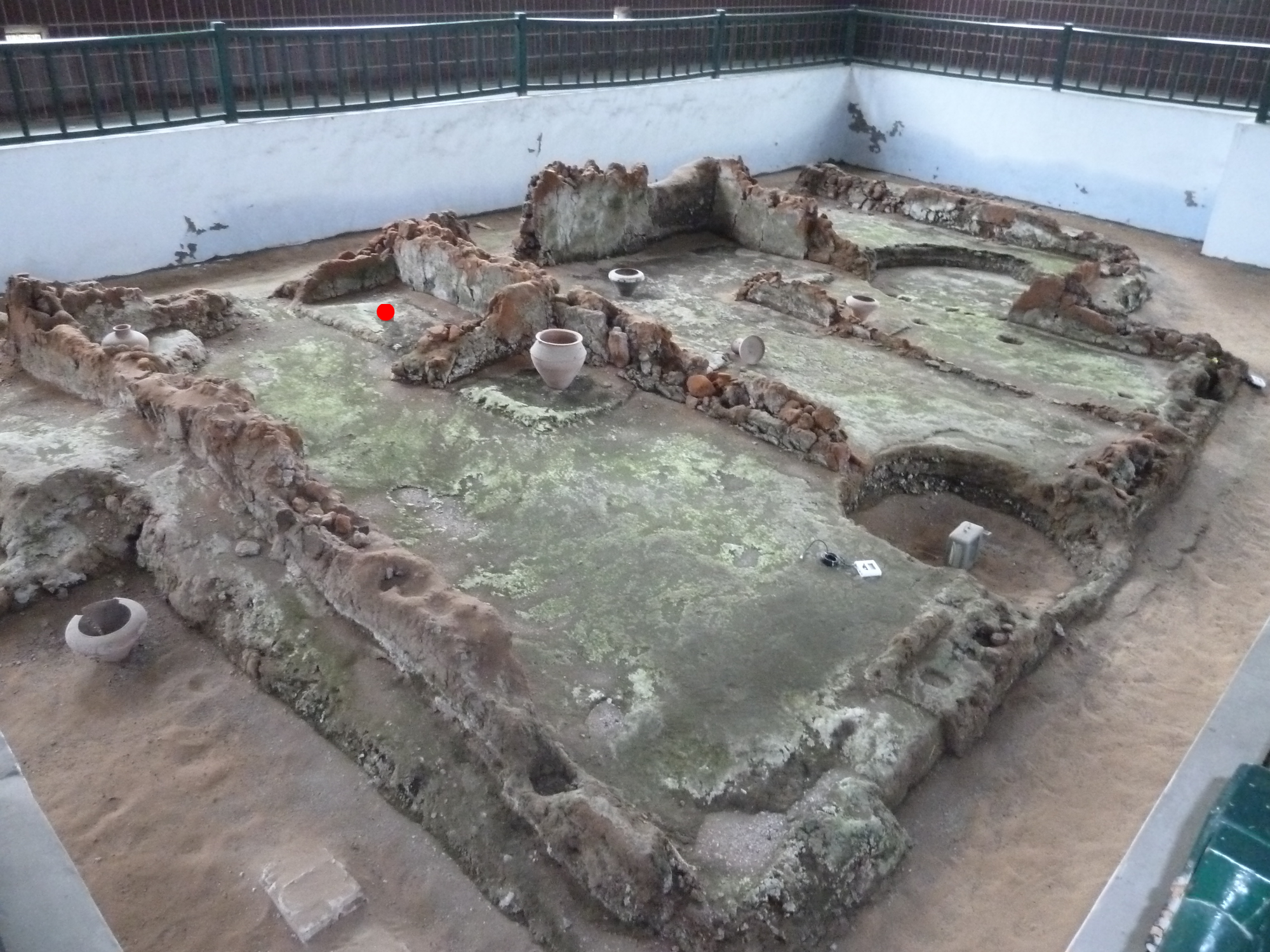

Supplement: Figure S1 — Yangshao Houses at the Dahecun site, Henan, China. Remains of houses at Dahecun, from left to right: F2, F1, F3. The pottery jar containing soybeans was discovered on the northeast corner of F2, indicated with a red dot. The jar however is now misplaced in the central area near the eastern wall (photo taken by Li Liu at the Dahecun Museum, Zhengzhou, 2010). (TIF) [file pone.0026720.s001.tif]

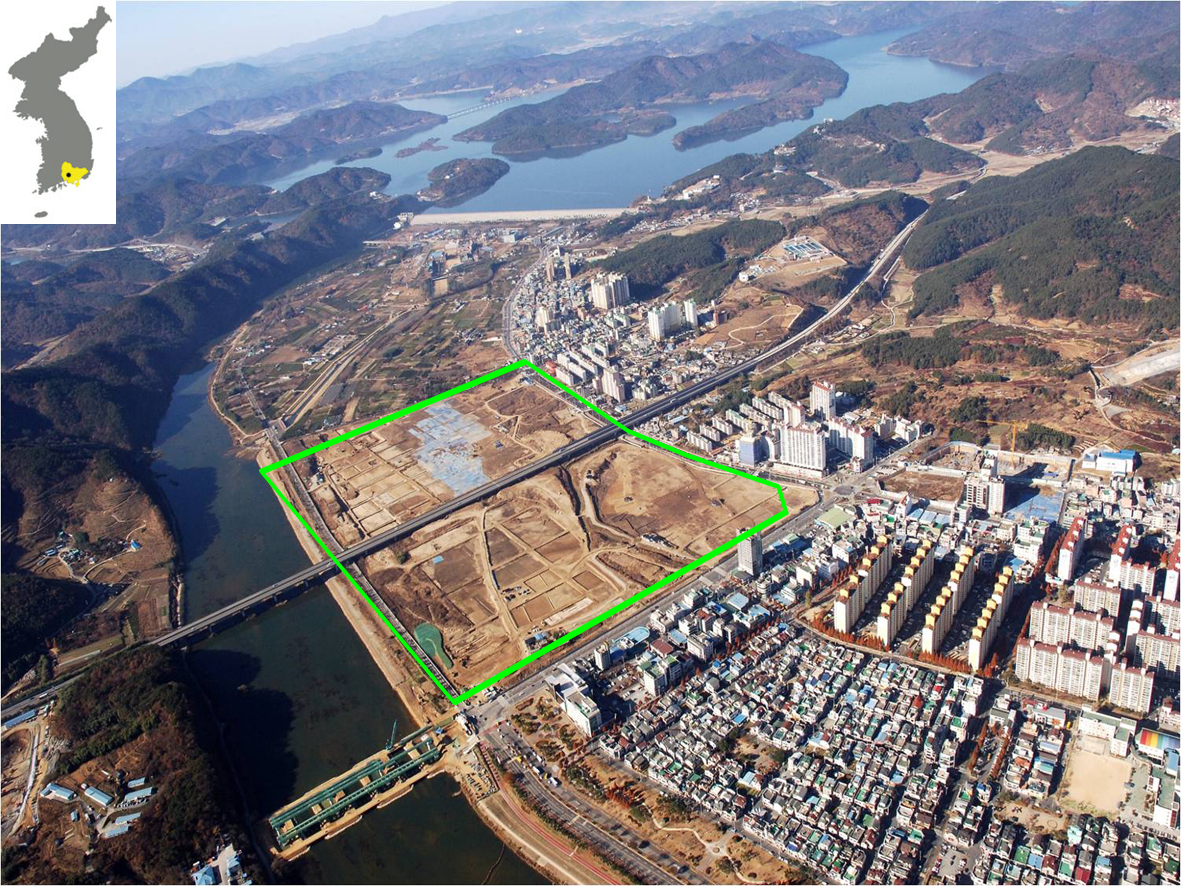

Supplement: Figure S2 — Aerial photograph of the Chulmun Pyeonggeodong site, Korea. Pyeonggeodong (Figure 2) is a multi-component site, situated in alluvial flats along the Nam River, southeastern Korea (highlighted in the inset). The area discussed is within the green boundary. Numerous structures, including pit houses, dolmen burials, farming fields, and hunting traps were recovered in an extensive area of 15 ha, dating from 5000 to 1200 BP. Analysis of plant remains from these features is ongoing. Most soybean specimens were found in Chulmun pits, including the one in Figure S3. (TIF) [file pone.0026720.s002.tif]

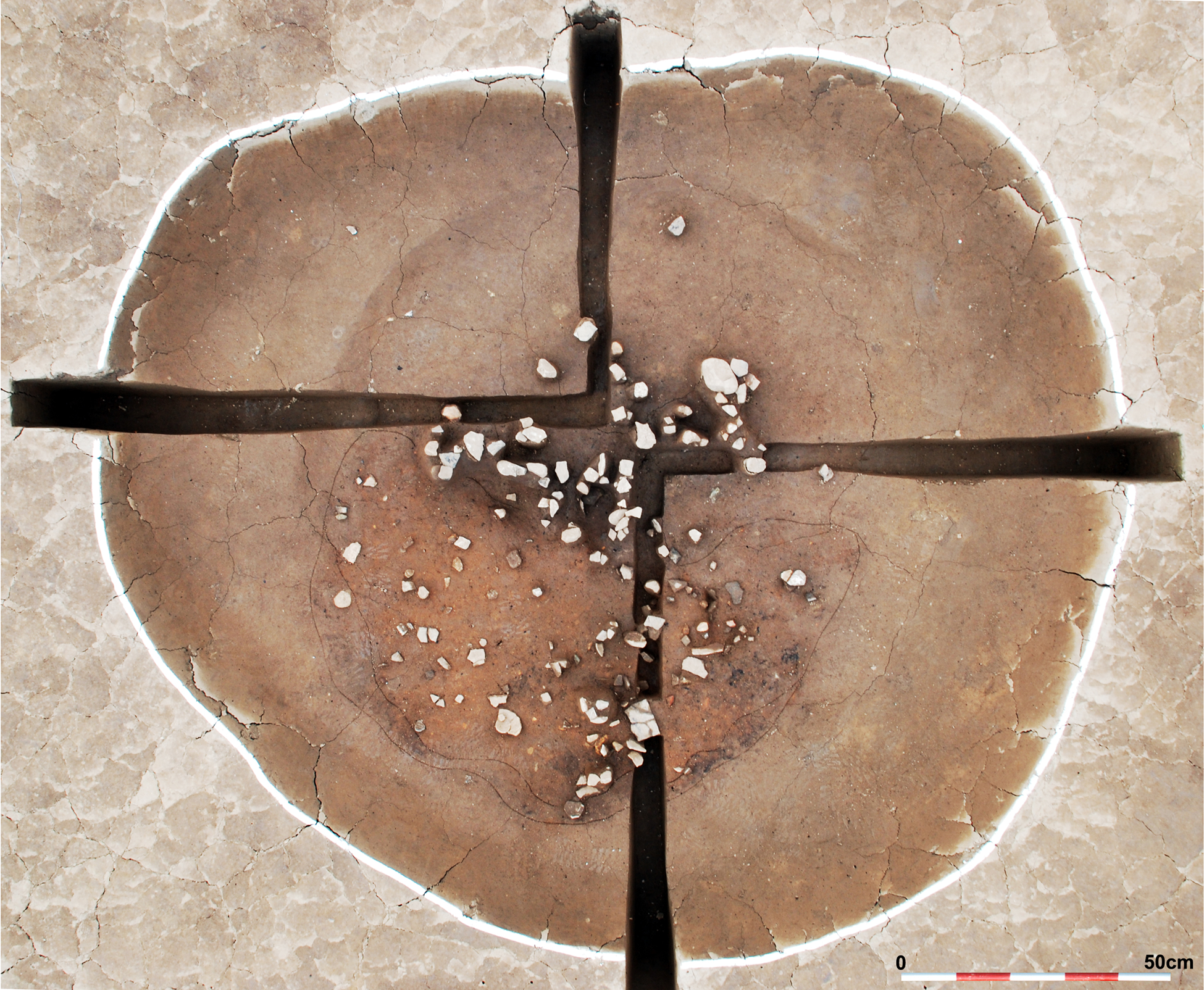

Supplement: Figure S3 — Chulmun pit at the Pyeonggeodong site. The pit feature (no. 28) contained charred soybean seeds along with azuki beans that are AMS-dated to 4830–4650 cal. BP (UCI60749, Table 1). (TIF) [file pone.0026720.s003.tif]

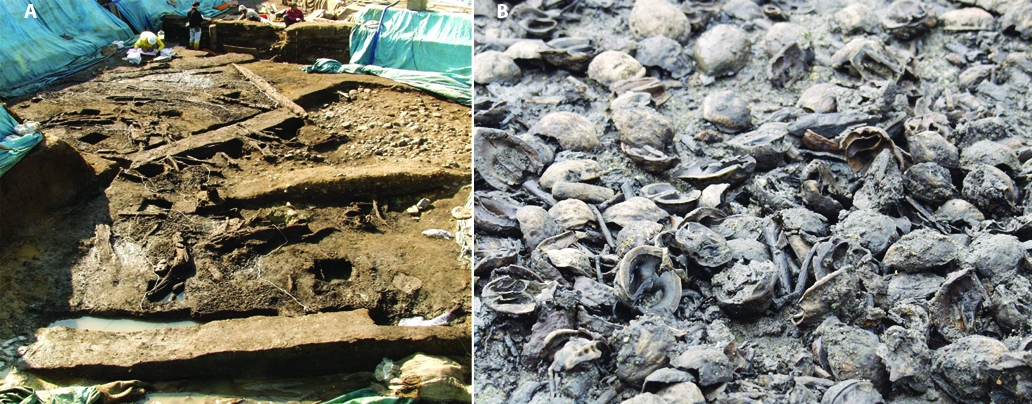

Supplement: Figure S4 — The Shimoyakebe site, Japan. A. Overview of the waterlogged, Middle Jomon walnut midden with well preserved wood; B. detail of walnut midden from where soybean sample was recovered. (TIF) [file pone.0026720.s004.tif]

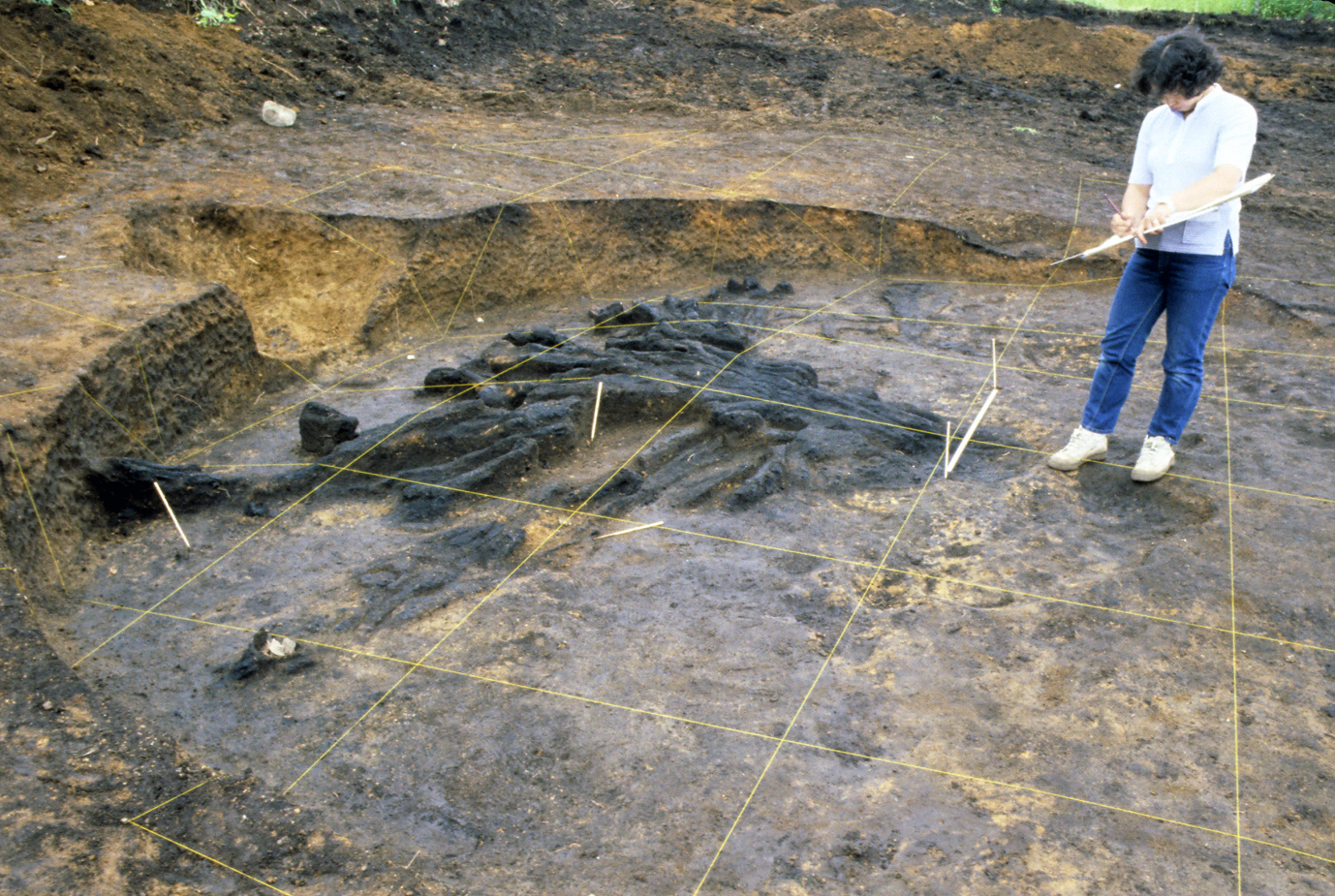

Supplement: Figure S5 — Pit House at Late Jomon Usujiri Shogakko site, Japan. Several of the pit houses excavated in 1977 at Ususjiri Shogakko were destroyed by fire. Parts of the charred superstructure lie on the floor. Flotation samples were collected from the floor among the charred wood fragments. (TIF) [file pone.0026720.s005.tif]
