# Supplementary material for: Archaeological Soybean (Glycine max) in East Asia: Does Size Matter?
Source: PLoS One. 2011 Nov 4;6(11):e26720. doi: 10.1371/journal.pone.0026720 (PMC3208558; doi:10.1371/journal.pone.0026720)
Supplement: Table S1 — Soybean seed sizes from the research area. (DOCX) [file pone.0026720.s006.docx]

**Supporting Information**

**Table S1.** Soybean seed sizes from the research area.

| **Sample** | **Accession N or Period/Site** | **Length** | **Width** | **Thickness** |
| --- | --- | --- | --- | --- |
| Modern domesticated *Glycine max* ssp.  *max* | IT209387 | 2.6 | 2.5 | 4.1 |
| Modern domesticated *Glycine max* ssp.  *max* | IT209387 | 3.0 | 2.8 | 4.6 |
| Modern domesticated *Glycine max* ssp.  *max* | IT209387 | 3.6 | 3.2 | 5.3 |
| Modern domesticated *Glycine max* ssp.  *max* | IT209387 | 2.7 | 2.4 | 4.3 |
| Modern domesticated *Glycine max* ssp.  *max* | IT209387 | 3.4 | 3.0 | 4.7 |
| Modern domesticated *Glycine max* ssp.  *max* | IT209387 | 4.5 | 4.7 | 4.4 |
| Modern domesticated *Glycine max* ssp.  *max* | IT209387 | 5.7 | 5.2 | 4.8 |
| Modern domesticated *Glycine max* ssp.  *max* | IT209387 | 6.6 | 6.0 | 5.4 |
| Modern domesticated *Glycine max* ssp.  *max* | IT209387 | 6.6 | 4.3 | 4.0 |
| Modern domesticated *Glycine max* ssp.  *max* | IT209387 | 5.8 | 5.1 | 4.6 |
| Modern domesticated *Glycine max* ssp.  *max* | IT209387 | 6.9 | 6.3 | 5.4 |
| Modern domesticated *Glycine max* ssp.  *max* | IT209387 | 6.7 | 6.2 | 5.4 |
| Modern domesticated *Glycine max* ssp.  *max* | IT209387 | 7.5 | 5.6 | 4.4 |
| Modern domesticated *Glycine max* ssp.  *max* | IT209387 | 6.2 | 5.6 | 5.0 |
| Modern domesticated *Glycine max* ssp.  *max* | IT209387 | 5.9 | 6.2 | 5.4 |
| Modern domesticated *Glycine max* ssp.  *max* | IT209387 | 5.9 | 5.4 | 4.8 |
| Modern domesticated *Glycine max* ssp.  *max* | IT209387 | 6.2 | 6.1 | 4.9 |
| Modern domesticated *Glycine max* ssp.  *max* | IT209387 | 6.2 | 5.5 | 5.4 |
| Modern domesticated *Glycine max* ssp.  *max* | IT209387 | 5.7 | 5.0 | 4.8 |
| Modern domesticated *Glycine max* ssp.  *max* | IT209387 | 6.0 | 5.3 | 4.6 |
| Modern domesticated *Glycine max* ssp.  *max* | IT209387 | 5.8 | 5.2 | 4.7 |
| Modern domesticated *Glycine max* ssp.  *max* | IT209387 | 5.7 | 5.4 | 4.5 |
| Modern domesticated *Glycine max* ssp.  *max* | IT209387 | 6.1 | 5.5 | 5.0 |
| Modern domesticated *Glycine max* ssp.  *max* | IT209387 | 5.9 | 5.3 | 5.0 |
| Modern domesticated *Glycine max* ssp.  *max* | IT209387 | 5.9 | 5.8 | 4.9 |
| Modern domesticated *Glycine max* ssp.  *max* | IT209387 | 6.0 | 5.1 | 5.2 |
| Modern domesticated *Glycine max* ssp.  *max* | IT209387 | 5.6 | 5.3 | 4.5 |
| Modern domesticated *Glycine max* ssp.  *max* | IT209387 | 5.6 | 5.3 | 5.1 |
| Modern domesticated *Glycine max* ssp.  *max* | IT209387 | 5.9 | 4.9 | 4.7 |
| Modern domesticated *Glycine max* ssp.  *max* | IT209387 | 2.6 | 2.5 | 4.4 |
| Modern wild *Glycine max* ssp. *soya* | IT822966 | 4.0 | 2.7 | 2.6 |
| Modern wild *Glycine max* ssp. *soya* | IT822966 | 4.1 | 3.0 | 2.4 |
| Modern wild *Glycine max* ssp. *soya* | IT822966 | 4.0 | 2.7 | 2.9 |
| Modern wild *Glycine max* ssp. *soya* | IT822966 | 3.9 | 2.7 | 2.4 |
| Modern wild *Glycine max* ssp. *soya* | IT822966 | 3.7 | 2.9 | 2.0 |
| Modern wild *Glycine max* ssp. *soya* | IT822966 | 3.5 | 2.4 | 2.7 |
| Modern wild *Glycine max* ssp. *soya* | IT822966 | 4.3 | 3.3 | 2.3 |
| Modern wild *Glycine max* ssp. *soya* | IT822966 | 4.1 | 2.6 | 2.3 |
| Modern wild *Glycine max* ssp. *soya* | IT822966 | 4.3 | 2.8 | 2.4 |
| Modern wild *Glycine max* ssp. *soya* | IT822966 | 3.5 | 2.3 | 2.0 |
| Modern wild *Glycine max* ssp. *soya* | IT822966 | 4.4 | 3.1 | 2.7 |
| Modern wild *Glycine max* ssp. *soya* | IT822966 | 4.0 | 3.4 | 2.8 |
| Modern wild *Glycine max* ssp. *soya* | IT822966 | 4.5 | 3.0 | 2.8 |
| Modern wild *Glycine max* ssp. *soya* | IT822966 | 4.3 | 2.8 | 2.6 |
| Modern wild *Glycine max* ssp. *soya* | IT822966 | 3.3 | 2.5 | 2.3 |
| Modern wild *Glycine max* ssp. *soya* | IT822966 | 4.1 | 2.9 | 2.3 |
| Modern wild *Glycine max* ssp. *soya* | IT822966 | 4.4 | 2.8 | 2.5 |
| Modern wild *Glycine max* ssp. *soya* | IT822966 | 4.4 | 3.1 | 2.7 |
| Modern wild *Glycine max* ssp. *soya* | IT822966 | 3.7 | 2.2 | 2.2 |
| Modern wild *Glycine max* ssp. *soya* | IT822966 | 4.3 | 3.0 | 2.7 |
| Modern wild *Glycine max* ssp. *soya* | IT822966 | 3.9 | 2.6 | 2.4 |
| Modern wild *Glycine max* ssp. *soya* | IT822966 | 3.5 | 2.5 | 1.6 |
| Modern wild *Glycine max* ssp. *soya* | IT822966 | 3.9 | 3.0 | 2.3 |
| Modern wild *Glycine max* ssp. *soya* | IT822966 | 4.3 | 3.2 | 2.8 |
| Modern wild *Glycine max* ssp. *soya* | IT822966 | 3.6 | 2.8 | 2.4 |
| Modern wild *Glycine max* ssp. *soya* | IT822966 | 4.6 | 2.3 | 2.2 |
| Modern wild *Glycine max* ssp. *soya* | IT822966 | 4.7 | 3.0 | 2.5 |
| Modern wild *Glycine max* ssp. *soya* | IT822966 | 4.4 | 2.7 | 2.5 |
| Modern wild *Glycine max* ssp. *soya* | IT822966 | 4.0 | 2.3 | 2.0 |
| Modern wild *Glycine max* ssp. *soya* | IT822966 | 3.9 | 2.8 | 2.3 |
| Modern wild *Glycine max* ssp. *soya* | IT822967 | 4.4 | 3.0 | 2.0 |
| Modern wild *Glycine max* ssp. *soya* | IT822967 | 5.0 | 3.2 | 2.9 |
| Modern wild *Glycine max* ssp. *soya* | IT822967 | 4.5 | 3.4 | 2.9 |
| Modern wild *Glycine max* ssp. *soya* | IT822967 | 4.5 | 2.8 | 2.2 |
| Modern wild *Glycine max* ssp. *soya* | IT822967 | 4.8 | 3.2 | 2.9 |
| Modern wild *Glycine max* ssp. *soya* | IT822967 | 4.2 | 3.0 | 2.5 |
| Modern wild *Glycine max* ssp. *soya* | IT822967 | 4.2 | 3.1 | 2.7 |
| Modern wild *Glycine max* ssp. *soya* | IT822967 | 3.7 | 2.9 | 2.6 |
| Modern wild *Glycine max* ssp. *soya* | IT822967 | 4.4 | 2.9 | 2.7 |
| Modern wild *Glycine max* ssp. *soya* | IT822967 | 3.7 | 2.8 | 2.0 |
| Modern wild *Glycine max* ssp. *soya* | IT822967 | 4.5 | 3.3 | 2.4 |
| Modern wild *Glycine max* ssp. *soya* | IT822967 | 4.0 | 3.1 | 2.4 |
| Modern wild *Glycine max* ssp. *soya* | IT822967 | 4.6 | 3.3 | 2.7 |
| Modern wild *Glycine max* ssp. *soya* | IT822967 | 4.3 | 3.1 | 2.5 |
| Modern wild *Glycine max* ssp. *soya* | IT822967 | 4.5 | 2.7 | 2.5 |
| Modern wild *Glycine max* ssp. *soya* | IT822967 | 4.6 | 3.3 | 2.5 |
| Modern wild *Glycine max* ssp. *soya* | IT822967 | 4.9 | 3.4 | 2.7 |
| Modern wild *Glycine max* ssp. *soya* | IT822967 | 4.1 | 3.0 | 2.1 |
| Modern wild *Glycine max* ssp. *soya* | IT822967 | 4.8 | 3.4 | 2.7 |
| Modern wild *Glycine max* ssp. *soya* | IT822967 | 4.4 | 3.4 | 2.7 |
| Modern wild *Glycine max* ssp. *soya* | IT822967 | 4.1 | 2.6 | 1.9 |
| Modern wild *Glycine max* ssp. *soya* | IT822967 | 4.9 | 3.5 | 2.9 |
| Modern wild *Glycine max* ssp. *soya* | IT822967 | 4.1 | 2.9 | 2.2 |
| Modern wild *Glycine max* ssp. *soya* | IT822967 | 4.8 | 3.5 | 2.9 |
| Modern wild *Glycine max* ssp. *soya* | IT822967 | 4.3 | 2.7 | 2.3 |
| Modern wild *Glycine max* ssp. *soya* | IT822967 | 4.1 | 3.2 | 2.3 |
| Modern wild *Glycine max* ssp. *soya* | IT822967 | 4.8 | 2.7 | 2.5 |
| Modern wild *Glycine max* ssp. *soya* | IT822967 | 4.1 | 2.8 | 1.9 |
| Modern wild *Glycine max* ssp. *soya* | IT822967 | 4.2 | 3.1 | 2.6 |
| Modern wild *Glycine max* ssp. *soya* | IT822967 | 5.0 | 3.0 | 2.0 |
| Modern wild *Glycine max* ssp. *soya* | Modern/Huizui | 3.0 | 2.1 | 1.6 |
| Modern wild *Glycine max* ssp. *soya* | Modern/Huizui | 3.0 | 2.3 | 1.7 |
| Modern wild *Glycine max* ssp. *soya* | Modern/Huizui | 2.9 | 2.1 | 1.5 |
| Modern wild *Glycine max* ssp. *soya* | Modern/Huizui | 2.4 | 1.9 | 1.8 |
| Modern wild *Glycine max* ssp. *soya* | Modern/Huizui | 2.9 | 2.3 | 1.7 |
| Modern wild *Glycine max* ssp. *soya* | Modern/Huizui | 2.8 | 2.2 | 2.1 |
| Modern wild *Glycine max* ssp. *soya* | Modern/Huizui | 2.9 | 2.1 | 1.8 |
| Modern wild *Glycine max* ssp. *soya* | Modern/Huizui | 2.9 | 2.2 | 1.6 |
| Modern wild *Glycine max* ssp. *soya* | Modern/Huizui | 2.5 | 2.2 | 1.7 |
| Modern wild *Glycine max* ssp. *soya* | Modern/Huizui | 2.4 | 2.0 | 1.9 |
| Modern wild *Glycine max* ssp. *soya* | Modern/Huizui | 3.0 | 2.2 | 1.7 |
| Modern wild *Glycine max* ssp. *soya* | Modern/Huizui | 2.8 | 2.0 | 1.9 |
| Modern wild *Glycine max* ssp. *soya* | Modern/Huizui | 2.9 | 2.1 | 1.8 |
| Modern wild *Glycine max* ssp. *soya* | Modern/Huizui | 2.8 | 2.0 | 1.7 |
| Modern wild *Glycine max* ssp. *soya* | Modern/Huizui | 2.7 | 2.1 | 1.5 |
| Modern wild *Glycine max* ssp. *soya* | Modern/Huizui | 3.0 | 1.9 | 1.6 |
| Modern wild *Glycine max* ssp. *soya* | Modern/Huizui | 2.3 | 2.0 | 2.0 |
| Modern wild *Glycine max* ssp. *soya* | Modern/Huizui | 2.8 | 2.2 | 1.5 |
| Modern wild *Glycine max* ssp. *soya* | Modern/Huizui | 3.1 | 2.1 | 1.6 |
| Modern wild *Glycine max* ssp. *soya* | Modern/Huizui | 2.4 | 2.0 | 1.5 |
| Modern wild *Glycine max* ssp. *soya* | Modern/Huizui | 2.7 | 2.1 | 1.4 |
| Modern wild *Glycine max* ssp. *soya* | Modern/Huizui | 2.8 | 2.2 | 1.7 |
| Modern wild *Glycine max* ssp. *soya* | Modern/Huizui | 3.0 | 2.3 | 1.7 |
| Modern wild *Glycine max* ssp. *soya* | Modern/Huizui | 2.8 | 2.0 | 1.8 |
| Modern wild *Glycine max* ssp. *soya* | Modern/Huizui | 2.7 | 2.2 | 1.9 |
| Modern wild *Glycine max* ssp. *soya* | Modern/Huizui | 2.5 | 2.1 | 1.4 |
| Modern wild *Glycine max* ssp. *soya* | Modern/Huizui | 2.9 | 2.3 | 1.6 |
| Modern wild *Glycine max* ssp. *soya* | Modern/Huizui | 2.9 | 2.2 | 1.7 |
| Modern wild *Glycine max* ssp. *soya* | Modern/Huizui | 2.9 | 2.4 | 2.0 |
| Modern wild *Glycine max* ssp. *soya* | Modern/Huizui | 2.9 | 2.3 | 1.9 |
| Charred *Glycine max* | Early Neolithic/Jiahu | 2.8 | 2.3 |  |
| Charred *Glycine max* | Early Neolithic/Jiahu | 2.5 | 2.0 |  |
| Charred *Glycine max* | Early Neolithic/Jiahu | 3.0 | 2.1 |  |
| Charred *Glycine max* | Early Neolithic/Jiahu | 2.7 | 1.7 |  |
| Charred *Glycine max* | Early Neolithic/Jiahu | 2.9 | 2.4 |  |
| Charred *Glycine max* | Early Neolithic/Jiahu | 3.3 | 1.9 |  |
| Charred *Glycine max* | Early Neolithic/Jiahu | 3.0 | 1.9 |  |
| Charred *Glycine max* | Early Neolithic/Jiahu | 2.8 | 2.1 |  |
| Charred *Glycine max* | Early Neolithic/Jiahu | 2.5 | 1.9 |  |
| Charred *Glycine max* | Early Neolithic/Jiahu | 2.4 | 1.5 |  |
| Charred *Glycine max* | Early Neolithic/Jiahu | 2.3 | 1.6 |  |
| Charred *Glycine max* | Early Neolithic/Jiahu | 1.7 | 1.4 |  |
| Charred *Glycine max* | Early Neolithic/Jiahu | 2.1 | 1.3 |  |
| Charred *Glycine max* | Early Neolithic/Jiahu | 2.1 | 1.3 |  |
| Charred *Glycine max* | Early Neolithic/Jiahu | 2.2 | 1.6 |  |
| Charred *Glycine max* | Early Neolithic/Jiahu | 2.2 | 1.3 |  |
| Charred *Glycine max* | Early Neolithic/Jiahu | 2.1 | 1.5 |  |
| Charred *Glycine max* | Early Neolithic/Jiahu | 2.2 | 1.4 |  |
| Charred *Glycine max* | Early Neolithic/Jiahu | 2.1 | 1.4 |  |
| Charred *Glycine max* | Early Neolithic/Jiahu | 2.4 | 2.0 |  |
| Charred *Glycine max* | Early Neolithic/Jiahu | 2.5 | 1.4 |  |
| Charred *Glycine max* | Early Neolithic/Jiahu | 2.0 | 1.4 |  |
| Charred *Glycine max* | Early Neolithic/Jiahu | 2.4 | 1.3 |  |
| Charred *Glycine max* | Early Neolithic/Jiahu | 2.6 | 1.4 |  |
| Charred *Glycine max* | Early Neolithic/Jiahu | 2.7 | 1.4 |  |
| Charred *Glycine max* | Early Neolithic/Jiahu | 2.1 | 1.5 |  |
| Charred *Glycine max* | Early Neolithic/Jiahu | 1.9 | 1.5 |  |
| Charred *Glycine max* | Early Neolithic/Jiahu | 1.7 | 1.4 |  |
| Charred *Glycine max* | Early Neolithic/Jiahu | 1.7 | 1.3 |  |
| Charred *Glycine max* | Early Neolithic/Jiahu | 1.8 | 1.2 |  |
| Charred *Glycine max* | Early Neolithic/Jiahu | 1.7 | 1.3 |  |
| Charred *Glycine max* | Early Neolithic/Jiahu | 1.9 | 1.3 |  |
| Charred *Glycine max* | Early Neolithic/Jiahu | 2.0 | 1.5 |  |
| Charred *Glycine max* | Early Neolithic/Jiahu | 1.8 | 1.2 |  |
| Charred *Glycine max* | Early Neolithic/Jiahu | 3.2 | 2.7 |  |
| Charred *Glycine max* | Early Neolithic/Jiahu | 3.0 | 2.3 |  |
| Charred *Glycine max* | Early Neolithic/Jiahu | 3.4 | 2.4 |  |
| Charred *Glycine max* | Early Neolithic/Jiahu | 3.2 | 2.2 |  |
| Charred *Glycine max* | Early Neolithic/Jiahu | 2.7 | 2.3 |  |
| Charred *Glycine max* | Early Neolithic/Jiahu | 2.6 | 1.8 |  |
| Charred *Glycine max* | Early Neolithic/Jiahu | 3.1 | 2.5 |  |
| Charred *Glycine max* | Early Neolithic/Jiahu | 2.8 | 1.9 |  |
| Charred *Glycine max* | Early Neolithic/Jiahu | 3.5 | 2.3 |  |
| Charred *Glycine max* | Early Neolithic/Jiahu | 3.2 | 2.2 |  |
| Charred *Glycine max* | Early Neolithic/Jiahu | 3.0 | 1.9 |  |
| Charred *Glycine max* | Early Neolithic/Jiahu | 3.2 | 2.1 |  |
| Charred *Glycine max* | Early Neolithic/Jiahu | 2.7 | 2.2 |  |
| Charred *Glycine max* | Early Neolithic/Jiahu | 2.8 | 1.7 |  |
| Charred *Glycine max* | Early Neolithic/Jiahu | 3.6 | 2.3 | 1.8 |
| Charred *Glycine max* | Early Neolithic/Jiahu | 4.0 | 2.0 | 2.0 |
| Charred *Glycine max* | Early Neolithic/Jiahu | 3.0 | 2.4 | 2.2 |
| Charred *Glycine max* | Early Neolithic/Jiahu | 2.8 | 2.2 | 1.8 |
| Charred *Glycine max* | Early Neolithic/Jiahu | 2.8 | 1.9 | 1.8 |
| Charred *Glycine max* | Early Neolithic/Jiahu | 2.8 | 1.9 | 1.7 |
| Charred *Glycine max* | Early Neolithic/Jiahu | 3.3 | 2.4 | 1.8 |
| Charred *Glycine max* | Early Neolithic/Jiahu | 2.7 | 2.0 | 1.7 |
| Charred *Glycine max* | Early Neolithic/Jiahu | 3.0 | 2.0 | 1.8 |
| Charred *Glycine max* | Early Neolithic/Jiahu | 2.8 | 2.1 | 2.5 |
| Charred *Glycine max* | Early Neolithic/Jiahu | 2.8 | 1.8 | 2.0 |
| Charred *Glycine max* | Early Neolithic/Jiahu | 2.8 | 1.9 | 1.9 |
| Charred *Glycine max* | Early Neolithic/Jiahu | 4.2 | 3.1 | 2.5 |
| Charred *Glycine max* | Early Neolithic/Jiahu | 3.6 | 2.3 | 2.4 |
| Charred *Glycine max* | Early Neolithic/Jiahu | 3.4 | 2.5 | 2.3 |
| Charred *Glycine max* | Early Neolithic/Jiahu | 3.0 | 2.0 | 2.0 |
| Charred *Glycine max* | Early Neolithic/Jiahu | 3.4 | 2.1 | 2.1 |
| Charred *Glycine max* | Early Neolithic/Jiahu | 3.3 | 2.2 | 1.9 |
| Charred *Glycine max* | Early Neolithic/Jiahu | 3.2 | 2.0 | 2.1 |
| Charred *Glycine max* | Early Neolithic/Jiahu | 3.2 | 1.9 | 1.9 |
| Charred *Glycine max* | Early Neolithic/Jiahu | 2.8 | 1.8 | 2.0 |
| Charred *Glycine max* | Early Neolithic/Jiahu | 3.0 | 2.1 | 1.8 |
| Charred *Glycine max* | Early Neolithic/Jiahu | 3.2 | 1.9 | 1.6 |
| Charred *Glycine max* | Early Neolithic/Jiahu | 3.0 | 2.3 | 1.8 |
| Charred *Glycine max* | Early Neolithic/Jiahu | 3.4 | 2.3 | 1.8 |
| Charred *Glycine max* | Early Neolithic/Jiahu | 2.7 | 2.1 | 1.6 |
| Charred *Glycine max* | Early Neolithic/Jiahu | 2.9 | 2.3 | 2.1 |
| Charred *Glycine max* | Early Neolithic/Jiahu | 3.6 | 2.5 | 2.1 |
| Charred *Glycine max* | Early Neolithic/Jiahu | 3.0 | 1.9 | 1.6 |
| Charred *Glycine max* | Early Neolithic/Jiahu | 3.1 | 2.5 | 2.2 |
| Charred *Glycine max* | Early Neolithic/Jiahu | 4.9 | 3.4 | 3.0 |
| Charred *Glycine max* | Early Neolithic/Jiahu | 3.6 | 2.7 | 2.0 |
| Charred *Glycine max* | Early Neolithic/Jiahu | 3.2 | 2.5 | 2.1 |
| Charred *Glycine max* | Early Neolithic/Jiahu | 3.3 | 2.0 | 2.2 |
| Charred *Glycine max* | Early Neolithic/Jiahu | 3.8 | 2.2 | 2.0 |
| Charred *Glycine max* | Early Neolithic/Jiahu | 3.1 | 2.1 | 2.2 |
| Charred *Glycine max* | Early Neolithic/Jiahu | 4.6 | 3.2 | 2.0 |
| Charred *Glycine max* | Early Neolithic/Jiahu | 3.4 | 2.5 | 1.9 |
| Charred *Glycine max* | Early Neolithic/Jiahu | 3.8 | 2.2 | 1.9 |
| Charred *Glycine max* | Early Neolithic/Jiahu | 3.2 | 2.3 | 2.0 |
| Charred *Glycine max* | Early Neolithic/Jiahu | 3.0 | 2.2 | 2.3 |
| Charred *Glycine max* | Early Neolithic/Jiahu | 3.0 | 2.2 | 1.8 |
| Charred *Glycine max* | Early Neolithic/Jiahu | 3.3 | 2.4 | 2.2 |
| Charred *Glycine max* | Early Neolithic/Jiahu | 3.6 | 2.4 | 2.0 |
| Charred *Glycine max* | Early Neolithic/Jiahu | 3.5 | 2.7 | 2.0 |
| Charred *Glycine max* | Early Neolithic/Jiahu | 3.5 | 2.8 | 1.6 |
| Charred *Glycine max* | Early Neolithic/Jiahu | 3.3 | 2.4 | 2.0 |
| Charred *Glycine max* | Early Neolithic/Jiahu | 3.3 | 2.5 | 1.8 |
| Charred *Glycine max* | Early Neolithic/Jiahu | 3.5 | 2.5 | 1.8 |
| Charred *Glycine max* | Early Neolithic/Jiahu | 3.9 | 2.8 | 1.8 |
| Charred *Glycine max* | Early Neolithic/Jiahu | 4.2 | 2.4 | 1.7 |
| Charred *Glycine max* | Early Neolithic/Jiahu | 3.0 | 2.3 | 2.0 |
| Charred *Glycine max* | Early Neolithic/Jiahu | 4.0 | 2.6 | 1.9 |
| Charred *Glycine max* | Early Neolithic/Jiahu | 3.3 | 2.3 | 1.9 |
| Charred *Glycine max* | Early Neolithic/Jiahu | 3.5 | 2.3 | 1.8 |
| Charred *Glycine max* | Early Neolithic/Jiahu | 3.6 | 2.5 | 1.6 |
| Charred *Glycine max* | Early Neolithic/Jiahu | 4.0 | 2.3 | 2.0 |
| Charred *Glycine max* | Early Neolithic/Jiahu | 3.2 | 2.4 | 2.0 |
| Charred *Glycine max* | Early Neolithic/Jiahu | 3.4 | 2.3 | 1.8 |
| Charred *Glycine max* | Early Neolithic/Jiahu | 3.5 | 2.5 | 2.0 |
| Charred *Glycine max* | Early Neolithic/Jiahu | 3.3 | 2.4 | 1.7 |
| Charred *Glycine max* | Early Neolithic/Jiahu | 3.8 | 2.3 | 1.8 |
| Charred *Glycine max* | Early Neolithic/Jiahu | 3.3 | 2.2 | 2.0 |
| Charred *Glycine max* | Early Neolithic/Jiahu | 3.4 | 2.2 | 1.8 |
| Charred *Glycine max* | Early Neolithic/Jiahu | 3.2 | 2.3 | 2.0 |
| Charred *Glycine max* | Early Neolithic/Jiahu | 3.1 | 2.3 | 1.6 |
| Charred *Glycine max* | Early Neolithic/Jiahu | 3.2 | 2.4 | 1.6 |
| Charred *Glycine max* | Early Neolithic/Jiahu | 3.3 | 2.0 | 1.7 |
| Charred *Glycine max* | Early Neolithic/Jiahu | 2.8 | 2.9 | 1.8 |
| Charred *Glycine max* | Early Neolithic/Jiahu | 2.8 | 1.8 | 1.5 |
| Charred *Glycine max* | Early Neolithic/Jiahu | 3.0 | 1.9 | 1.9 |
| Charred *Glycine max* | Early Neolithic/Jiahu | 3.5 | 2.4 | 1.6 |
| Charred *Glycine max* | Early Neolithic/Jiahu | 3.4 | 2.2 | 1.6 |
| Charred *Glycine max* | Early Neolithic/Jiahu | 3.0 | 1.9 | 1.9 |
| Charred *Glycine max* | Early Neolithic/Jiahu | 3.2 | 1.7 | 1.7 |
| Charred *Glycine max* | Early Neolithic/Jiahu | 3.3 | 2.4 | 1.8 |
| Charred *Glycine max* | Early Neolithic/Jiahu | 3.8 | 3.1 | 2.2 |
| Charred *Glycine max* | Early Neolithic/Jiahu | 3.1 | 2.6 | 2.0 |
| Charred *Glycine max* | Early Neolithic/Jiahu | 2.9 | 2.4 | 1.8 |
| Charred *Glycine max* | Early Neolithic/Jiahu | 3.3 | 2.5 | 2.0 |
| Charred *Glycine max* | Early Neolithic/Jiahu | 2.9 | 2.0 | 2.1 |
| Charred *Glycine max* | Early Neolithic/Jiahu | 2.8 | 2.3 | 1.8 |
| Charred *Glycine max* | Early Neolithic/Jiahu | 3.1 | 2.2 | 2.1 |
| Charred *Glycine max* | Early Neolithic/Jiahu | 2.8 | 2.0 | 1.9 |
| Charred *Glycine max* | Early Neolithic/Jiahu | 3.1 | 2.3 | 1.8 |
| Charred *Glycine max* | Early Neolithic/Jiahu | 3.1 | 2.1 | 2.3 |
| Charred *Glycine max* | Early Neolithic/Jiahu | 2.4 | 1.7 | 2.0 |
| Charred *Glycine max* | Early Neolithic/Jiahu | 2.7 | 1.5 | 2.3 |
| Charred *Glycine max* | Early Neolithic/Jiahu | 3.7 | 2.6 | 1.7 |
| Charred *Glycine max* | Early Neolithic/Jiahu | 3.9 | 2.8 | 1.8 |
| Charred *Glycine max* | Early Neolithic/Jiahu | 3.8 | 2.4 | 1.9 |
| Charred *Glycine max* | Early Neolithic/Jiahu | 3.4 | 2.2 | 2.6 |
| Charred *Glycine max* | Early Neolithic/Jiahu | 3.6 | 2.3 | 2.3 |
| Charred *Glycine max* | Early Neolithic/Jiahu | 3.4 | 2.2 | 2.2 |
| Charred *Glycine max* | Early Neolithic/Jiahu | 3.8 | 1.9 | 2.2 |
| Charred *Glycine max* | Early Neolithic/Jiahu | 3.2 | 2.3 | 2.0 |
| Charred *Glycine max* | Early Neolithic/Jiahu | 2.8 | 1.8 | 2.0 |
| Charred *Glycine max* | Early Neolithic/Jiahu | 2.7 | 2.0 | 1.7 |
| Charred *Glycine max* | Early Neolithic/Jiahu | 2.5 | 2.0 | 1.4 |
| Charred *Glycine max* | Early Neolithic/Jiahu | 2.6 | 1.5 | 1.5 |
| Charred *Glycine max* | Early Neolithic/Jiahu | 4.2 | 3.1 | 2.5 |
| Charred *Glycine max* | Early Neolithic/Jiahu | 3.1 | 2.4 | 2.0 |
| Charred *Glycine max* | Early Neolithic/Jiahu | 3.3 | 2.3 | 1.8 |
| Charred *Glycine max* | Early Neolithic/Jiahu | 3.3 | 2.2 | 1.7 |
| Charred *Glycine max* | Early Neolithic/Jiahu | 3.1 | 2.6 | 1.7 |
| Charred *Glycine max* | Early Neolithic/Jiahu | 2.8 | 2.4 | 1.7 |
| Charred *Glycine max* | Early Neolithic/Jiahu | 3.4 | 2.6 | 1.3 |
| Charred *Glycine max* | Early Neolithic/Jiahu | 3.2 | 2.1 | 1.1 |
| Charred *Glycine max* | Early Neolithic/Jiahu | 3.2 | 2.4 | 2.1 |
| Charred *Glycine max* | Early Neolithic/Jiahu | 4.4 | 2.9 | 2.4 |
| Charred *Glycine max* | Early Neolithic/Jiahu | 3.1 | 2.8 | 2.3 |
| Charred *Glycine max* | Early Neolithic/Jiahu | 3.2 | 2.2 | 2.1 |
| Charred *Glycine max* | Early Neolithic/Jiahu | 3.5 | 2.3 | 1.9 |
| Charred *Glycine max* | Early Neolithic/Jiahu | 3.8 | 2.8 | 2.2 |
| Charred *Glycine max* | Early Neolithic/Jiahu | 4.1 | 2.9 | 2.0 |
| Charred *Glycine max* | Early Neolithic/Jiahu | 3.9 | 3.1 | 2.4 |
| Charred *Glycine max* | Early Neolithic/Jiahu | 3.0 | 2.3 | 2.2 |
| Charred *Glycine max* | Early Neolithic/Jiahu | 3.2 | 2.5 | 2.3 |
| Charred *Glycine max* | Early Neolithic/Jiahu | 3.6 | 2.5 | 2.1 |
| Charred *Glycine max* | Early Neolithic/Jiahu | 3.5 | 2.3 | 2.2 |
| Charred *Glycine max* | Early Neolithic/Jiahu | 3.7 | 2.5 | 2.4 |
| Charred *Glycine max* | Early Neolithic/Jiahu | 2.5 | 2.2 | 2.2 |
| Charred *Glycine max* | Early Neolithic/Jiahu | 3.0 | 2.4 | 1.9 |
| Charred *Glycine max* | Early Neolithic/Jiahu | 2.5 | 2.7 | 2.1 |
| Charred *Glycine max* | Early Neolithic/Jiahu | 3.0 | 2.0 | 2.2 |
| Charred *Glycine max* | Early Neolithic/Jiahu | 3.1 | 2.4 | 2.2 |
| Charred *Glycine max* | Early Neolithic/Jiahu | 3.0 | 2.4 | 1.8 |
| Charred *Glycine max* | Early Neolithic/Jiahu | 2.9 | 2.3 | 2.0 |
| Charred *Glycine max* | Early Neolithic/Jiahu | 3.5 | 2.5 | 2.0 |
| Charred *Glycine max* | Early Neolithic/Jiahu | 3.1 | 2.3 | 1.9 |
| Charred *Glycine max* | Early Neolithic/Jiahu | 3.2 | 2.8 | 1.8 |
| Charred *Glycine max* | Early Neolithic/Jiahu | 3.6 | 3.0 | 1.9 |
| Charred *Glycine max* | Early Neolithic/Jiahu | 2.8 | 2.4 | 1.9 |
| Charred *Glycine max* | Early Neolithic/Jiahu | 3.5 | 2.1 | 1.9 |
| Charred *Glycine max* | Early Neolithic/Jiahu | 2.9 | 2.3 | 2.0 |
| Charred *Glycine max* | Early Neolithic/Jiahu | 2.9 | 1.8 | 1.8 |
| Charred *Glycine max* | Early Neolithic/Jiahu | 3.5 | 2.7 | 1.8 |
| Charred *Glycine max* | Early Neolithic/Jiahu | 2.9 | 2.3 | 2.0 |
| Charred *Glycine max* | Early Neolithic/Jiahu | 2.8 | 2.5 | 1.8 |
| Charred *Glycine max* | Early Neolithic/Jiahu | 3.8 | 2.4 | 2.4 |
| Charred *Glycine max* | Early Neolithic/Jiahu | 3.1 | 2.1 | 1.8 |
| Charred *Glycine max* | Early Neolithic/Jiahu | 2.7 | 2.2 | 1.8 |
| Charred *Glycine max* | Early Neolithic/Jiahu | 2.5 | 1.6 | 1.5 |
| Charred *Glycine max* | Early Neolithic/Jiahu | 3.7 | 2.4 | 2.0 |
| Charred *Glycine max* | Early Neolithic/Jiahu | 3.5 | 2.4 | 2.2 |
| Charred *Glycine max* | Early Neolithic/Jiahu | 3.5 | 2.8 | 2.1 |
| Charred *Glycine max* | Early Neolithic/Jiahu | 4.1 | 2.9 | 2.4 |
| Charred *Glycine max* | Early Neolithic/Jiahu | 4.1 | 3.1 | 2.1 |
| Charred *Glycine max* | Early Neolithic/Jiahu | 3.9 | 2.9 | 2.3 |
| Charred *Glycine max* | Early Neolithic/Jiahu | 3.5 | 2.8 | 2.3 |
| Charred *Glycine max* | Early Neolithic/Jiahu | 3.4 | 2.9 | 2.2 |
| Charred *Glycine max* | Early Neolithic/Jiahu | 3.9 | 3.1 | 1.9 |
| Charred *Glycine max* | Early Neolithic/Jiahu | 3.7 | 2.8 | 2.2 |
| Charred *Glycine max* | Early Neolithic/Jiahu | 3.9 | 2.5 | 2.3 |
| Charred *Glycine max* | Early Neolithic/Jiahu | 4.4 | 2.8 | 1.9 |
| Charred *Glycine max* | Early Neolithic/Jiahu | 3.2 | 2.3 | 2.0 |
| Charred *Glycine max* | Early Neolithic/Jiahu | 2.9 | 2.2 | 2.1 |
| Charred *Glycine max* | Early Neolithic/Jiahu | 3.6 | 2.4 | 2.0 |
| Charred *Glycine max* | Early Neolithic/Jiahu | 3.8 | 2.3 | 2.0 |
| Charred *Glycine max* | Early Neolithic/Jiahu | 3.3 | 2.3 | 1.6 |
| Charred *Glycine max* | Early Neolithic/Jiahu | 3.2 | 2.2 | 2.0 |
| Charred *Glycine max* | Early Neolithic/Jiahu | 2.9 | 1.9 | 1.7 |
| Charred *Glycine max* | Early Neolithic/Jiahu | 2.9 | 2.0 | 1.5 |
| Charred *Glycine max* | Early Neolithic/Jiahu | 2.6 | 2.0 | 1.5 |
| Charred *Glycine max* | Early Neolithic/Jiahu | 2.5 | 1.8 | 1.9 |
| Charred *Glycine max* | Early Neolithic/Jiahu | 2.7 | 2.3 | 2.1 |
| Charred *Glycine max* | Early Neolithic/Jiahu | 3.4 | 2.1 | 1.8 |
| Charred *Glycine max* | Early Neolithic/Jiahu | 2.4 | 2.1 | 1.8 |
| Charred *Glycine max* | Early Neolithic/Jiahu | 3.3 | 2.2 | 2.1 |
| Charred *Glycine max* | Early Neolithic/Jiahu | 3.3 | 2.2 | 2.6 |
| Charred *Glycine max* | Early Neolithic/Jiahu | 4.0 | 2.9 | 2.0 |
| Charred *Glycine max* | Early Neolithic/Jiahu | 4.5 | 2.8 | 2.3 |
| Charred *Glycine max* | Early Neolithic/Jiahu | 3.1 | 2.1 | 2.3 |
| Charred *Glycine max* | Early Neolithic/Jiahu | 3.3 | 2.4 | 1.7 |
| Charred *Glycine max* | Early Neolithic/Jiahu | 2.8 | 2.2 | 1.6 |
| Charred *Glycine max* | Early Neolithic/Jiahu | 3.2 | 2.2 | 1.9 |
| Charred *Glycine max* | Early Neolithic/Jiahu | 3.4 | 2.5 | 2.4 |
| Charred *Glycine max* | Early Neolithic/Jiahu | 2.4 | 1.9 | 1.8 |
| Charred *Glycine max* | Early Neolithic/Jiahu | 2.5 | 1.9 | 1.9 |
| Charred *Glycine max* | Early Neolithic/Jiahu | 2.8 | 2.0 | 3.0 |
| Charred *Glycine max* | Early Neolithic/Jiahu | 3.0 | 2.1 | 2.3 |
| Charred *Glycine max* | Early Neolithic/Jiahu | 2.9 | 2.3 | 3.0 |
| Charred *Glycine max* | Early Neolithic/Jiahu | 2.9 | 1.9 | 2.0 |
| Charred *Glycine max* | Early Neolithic/Jiahu | 2.8 | 2.1 | 2.0 |
| Charred *Glycine max* | Early Neolithic/Jiahu | 2.9 | 2.2 | 1.8 |
| Charred *Glycine max* | Early Neolithic/Jiahu | 2.8 | 1.9 | 1.9 |
| Charred *Glycine max* | Early Neolithic/Jiahu | 3.7 | 2.9 | 2.2 |
| Charred *Glycine max* | Early Neolithic/Jiahu | 3.4 | 2.4 | 2.0 |
| Charred *Glycine max* | Early Neolithic/Jiahu | 2.9 | 1.8 | 1.7 |
| Charred *Glycine max* | Early Neolithic/Jiahu | 2.9 | 2.2 | 2.1 |
| Charred *Glycine max* | Early Neolithic/Jiahu | 2.8 | 2.0 | 1.9 |
| Charred *Glycine max* | Early Neolithic/Jiahu | 4.3 | 3.0 | 2.2 |
| Charred *Glycine max* | Early Neolithic/Jiahu | 3.2 | 2.7 | 2.3 |
| Charred *Glycine max* | Early Neolithic/Jiahu | 3.5 | 2.0 | 2.1 |
| Charred *Glycine max* | Early Neolithic/Jiahu | 4.2 | 3.4 | 2.5 |
| Charred *Glycine max* | Early Neolithic/Jiahu | 4.2 | 2.3 | 2.4 |
| Charred *Glycine max* | Early Neolithic/Jiahu | 4.4 | 2.3 | 1.9 |
| Charred *Glycine max* | Early Neolithic/Jiahu | 3.2 | 2.4 | 2.1 |
| Charred *Glycine max* | Early Neolithic/Jiahu | 3.1 | 2.6 | 2.0 |
| Charred *Glycine max* | Early Neolithic/Jiahu | 3.1 | 2.5 | 1.9 |
| Charred *Glycine max* | Early Neolithic/Yuezhuang | 3.2 | 1.9 | 1.7 |
| Charred *Glycine max* | Early Neolithic/Yuezhuang | 3.1 | 2.2 | 1.0 |
| Charred *Glycine max* | Late Yangshao/Zhaocheng | 1.7 | 1.3 |  |
| Charred *Glycine max* | Late Yangshao/Zhaocheng | 1.6 | 0.9 |  |
| Charred *Glycine max* | Late Yangshao/Huizui | 3.4 | 2.2 |  |
| Charred *Glycine max* | Late Yangshao/Huizui | 1.4 | 0.9 |  |
| Charred *Glycine max* | Late Yangshao/Huizui | 1.5 | 1.0 |  |
| Charred *Glycine max* | Late Yangshao/Huizui | 3.5 | 2.5 | 2.8 |
| Charred *Glycine max* | Late Yangshao/Huizui | 3.2 | 2.4 |  |
| Charred *Glycine max* | Late Yangshao/Huizui | 1.4 | 0.9 |  |
| Charred *Glycine max* | Late Yangshao/Huizui | 1.6 | 1.2 |  |
| Charred *Glycine max* | Late Yangshao/Huizui | 3.0 | 2.4 | 2.1 |
| Charred *Glycine max* | Late Yangshao/Huizui | 1.2 | 0.8 |  |
| Charred *Glycine max* | Late Yangshao/Huizui | 1.0 | 0.8 |  |
| Charred *Glycine max* | Late Yangshao/Xipo | 2.9 | 2.1 |  |
| Charred *Glycine max* | Late Yangshao/Xipo | 2.1 | 1.7 |  |
| Charred *Glycine max* | Late Yangshao/Xipo | 1.7 | 1.1 | 0.8 |
| Charred *Glycine max* | Late Yangshao/Xipo | 1.6 | 0.9 | 0.7 |
| Charred *Glycine max* | Late Yangshao/Xipo | 3.1 | 2.0 | 2.0 |
| Charred *Glycine max* | Late Yangshao/Dahecun | 3.3 | 2.1 |  |
| Charred *Glycine max* | Late Yangshao/Dahecun | 3.2 | 2.4 |  |
| Charred *Glycine max* | Late Yangshao/Dahecun | 3.1 | 2.4 |  |
| Charred *Glycine max* | Late Yangshao Dahecun | 3.0 | 2.2 |  |
| Charred *Glycine max* | Late YangshaoDahecun | 2.9 | 2.4 |  |
| Charred *Glycine max* | Late Yangshao/Dahecun | 3.2 | 2.6 |  |
| Charred *Glycine max* | Late Yangshao/Dahecun | 3.4 | 1.9 |  |
| Charred *Glycine max* | Late Yangshao/Dahecun | 3.4 | 2.4 |  |
| Charred *Glycine max* | Late Yangshao/Dahecun | 2.4 | 1.9 |  |
| Charred *Glycine max* | Late Yangshao/Dahecun | 2.5 | 1.7 |  |
| Charred *Glycine max* | Late Yangshao/Dahecun | 3.1 | 2.5 |  |
| Charred *Glycine max* | Late Yangshao/Dahecun | 2.4 | 1.8 |  |
| Charred *Glycine max* | Late Yangshao/Dahecun | 3.0 | 2.1 |  |
| Charred *Glycine max* | Late Yangshao/Dahecun | 2.8 | 2.3 |  |
| Charred *Glycine max* | Late Yangshao/Dahecun | 3.1 | 2.8 |  |
| Charred *Glycine max* | Late Yangshao/Dahecun | 3.0 | 2.3 |  |
| Charred *Glycine max* | Late Yangshao/Dahecun | 2.9 | 2.5 |  |
| Charred *Glycine max* | Late Yangshao/Dahecun | 2.5 | 2.0 |  |
| Charred *Glycine max* | Late Yangshao/Dahecun | 3.0 | 2.3 |  |
| Charred *Glycine max* | Late Yangshao/Dahecun | 3.0 | 2.6 |  |
| Charred *Glycine max* | Late Yangshao/Dahecun | 2.3 | 2.2 |  |
| Charred *Glycine max* | Late Yangshao/Dahecun | 3.2 | 2.1 |  |
| Charred *Glycine max* | Late Yangshao/Dahecun | 2.8 | 2.0 |  |
| Charred *Glycine max* | Late Yangshao/Dahecun | 3.0 | 1.9 |  |
| Charred *Glycine max* | Late Yangshao/Dahecun | 3.4 | 2.4 |  |
| Charred *Glycine max* | Late Yangshao/Dahecun | 3.0 | 1.9 |  |
| Charred *Glycine max* | Late Yangshao/Dahecun | 3.0 | 2.5 |  |
| Charred *Glycine max* | Late Yangshao/Dahecun | 3.3 | 2.3 |  |
| Charred *Glycine max* | Late Yangshao/Dahecun | 3.0 | 2.5 |  |
| Charred *Glycine max* | Late Yangshao/Dahecun | 2.7 | 2.1 |  |
| Charred *Glycine max* | Late Yangshao/Dahecun | 3.1 | 2.2 |  |
| Charred *Glycine max* | Late Yangshao/Dahecun | 3.1 | 2.8 |  |
| Charred *Glycine max* | Late Yangshao/Dahecun | 3.1 | 2.1 |  |
| Charred *Glycine max* | Late Yangshao/Dahecun | 3.3 | 2.3 |  |
| Charred *Glycine max* | Late Yangshao/Dahecun | 3.2 | 2.5 |  |
| Charred *Glycine max* | Late Yangshao/Dahecun | 3.1 | 2.2 |  |
| Charred *Glycine max* | Late Yangshao/Dahecun | 3.4 | 2.6 |  |
| Charred *Glycine max* | Late Yangshao/Dahecun | 3.2 | 2.0 |  |
| Charred *Glycine max* | Late Yangshao/Dahecun | 2.6 | 2.0 |  |
| Charred *Glycine max* | Late Yangshao/Dahecun | 2.7 | 2.2 |  |
| Charred *Glycine max* | Late Yangshao/Dahecun | 2.9 | 2.2 |  |
| Charred *Glycine max* | Late Yangshao/Dahecun | 2.7 | 1.7 |  |
| Charred *Glycine max* | Late Yangshao/Dahecun | 2.4 | 2.3 |  |
| Charred *Glycine max* | Late Yangshao/Dahecun | 2.2 | 1.9 |  |
| Charred *Glycine max* | Late Yangshao/Dahecun | 2.7 | 2.0 |  |
| Charred *Glycine max* | Late Yangshao/Dahecun | 2.9 | 2.1 |  |
| Charred *Glycine max* | Late Yangshao/Dahecun | 2.5 | 2.2 |  |
| Charred *Glycine max* | Late Yangshao/Dahecun | 2.8 | 2.2 |  |
| Charred *Glycine max* | Late Yangshao/Dahecun | 3.0 | 1.7 |  |
| Charred *Glycine max* | Late Yangshao/Dahecun | 2.4 | 2.0 |  |
| Charred *Glycine max* | Late Yangshao/Dahecun | 2.4 | 2.2 |  |
| Charred *Glycine max* | Late Yangshao/Dahecun | 2.6 | 2.1 |  |
| Charred *Glycine max* | Late Yangshao/Dahecun | 2.9 | 2.3 |  |
| Charred *Glycine max* | Late Yangshao/Dahecun | 3.5 | 2.3 |  |
| Charred *Glycine max* | Late Yangshao/Dahecun | 3.1 | 1.9 |  |
| Charred *Glycine max* | Late Yangshao/Dahecun | 3.2 | 2.7 |  |
| Charred *Glycine max* | Late Yangshao/Dahecun | 2.7 | 2.1 |  |
| Charred *Glycine max* | Late Yangshao/Dahecun | 2.9 | 1.9 |  |
| Charred *Glycine max* | Late Yangshao/Dahecun | 2.8 | 2.4 |  |
| Charred *Glycine max* | Late Yangshao/Dahecun | 2.7 | 2.3 |  |
| Charred *Glycine max* | Late Yangshao/Dahecun | 2.8 | 2.5 |  |
| Charred *Glycine max* | Late Yangshao/Dahecun | 2.9 | 2.0 |  |
| Charred *Glycine max* | Late Yangshao/Dahecun | 3.1 | 2.5 |  |
| Charred *Glycine max* | Late Yangshao/Dahecun | 3.2 | 2.0 |  |
| Charred *Glycine max* | Late Yangshao/Dahecun | 3.2 | 2.6 |  |
| Charred *Glycine max* | Late Yangshao/Dahecun | 3.1 | 2.2 |  |
| Charred *Glycine max* | Late Yangshao/Dahecun | 2.7 | 1.8 |  |
| Charred *Glycine max* | Late Yangshao/Dahecun | 2.8 | 2.0 |  |
| Charred *Glycine max* | Late Yangshao/Dahecun | 3.0 | 1.8 |  |
| Charred *Glycine max* | Late Yangshao/Dahecun | 3.0 | 2.6 |  |
| Charred *Glycine max* | Late Yangshao/Dahecun | 2.9 | 2.1 |  |
| Charred *Glycine max* | Late Yangshao/Dahecun | 2.9 | 2.1 |  |
| Charred *Glycine max* | Late Yangshao/Dahecun | 2.9 | 2.3 |  |
| Charred *Glycine max* | Late Yangshao/Dahecun | 2.7 | 2.3 |  |
| Charred *Glycine max* | Late Yangshao/Dahecun | 3.0 | 1.9 |  |
| Charred *Glycine max* | Late Yangshao/Dahecun | 2.9 | 2.1 |  |
| Charred *Glycine max* | Late Yangshao/Dahecun | 2.6 | 1.9 |  |
| Charred *Glycine max* | Late Yangshao/Dahecun | 2.7 | 1.8 |  |
| Charred *Glycine max* | Late Yangshao/Dahecun | 3.3 | 2.9 |  |
| Charred *Glycine max* | Late Yangshao/Dahecun | 2.6 | 1.9 |  |
| Charred *Glycine max* | Late Yangshao/Dahecun | 3.1 | 2.1 |  |
| Charred *Glycine max* | Late Yangshao/Dahecun | 2.9 | 2.4 |  |
| Charred *Glycine max* | Late Yangshao/Dahecun | 2.7 | 2.2 |  |
| Charred *Glycine max* | Late Yangshao/Dahecun | 2.9 | 2.2 |  |
| Charred *Glycine max* | Late Yangshao/Dahecun | 2.7 | 2.3 |  |
| Charred *Glycine max* | Late Yangshao/Dahecun | 2.8 | 1.9 |  |
| Charred *Glycine max* | Late Yangshao/Dahecun | 3.6 | 2.7 |  |
| Charred *Glycine max* | Late Yangshao/Dahecun | 3.1 | 1.9 |  |
| Charred *Glycine max* | Late Yangshao/Dahecun | 2.7 | 2.2 |  |
| Charred *Glycine max* | Late Yangshao/Dahecun | 2.9 | 2.0 |  |
| Charred *Glycine max* | Late Yangshao/Dahecun | 2.8 | 2.0 |  |
| Charred *Glycine max* | Late Yangshao/Dahecun | 3.6 | 2.6 |  |
| Charred *Glycine max* | Late Yangshao/Dahecun | 3.1 | 2.4 |  |
| Charred *Glycine max* | Late Yangshao/Dahecun | 3.1 | 2.1 |  |
| Charred *Glycine max* | Late Yangshao/Dahecun | 3.3 | 2.5 |  |
| Charred *Glycine max* | Late Yangshao/Dahecun | 2.8 | 2.0 |  |
| Charred *Glycine max* | Late Yangshao/Dahecun | 2.9 | 2.2 |  |
| Charred *Glycine max* | Late Yangshao/Dahecun | 3.0 | 2.0 |  |
| Charred *Glycine max* | Late Yangshao/Dahecun | 4.2 | 2.6 |  |
| Charred *Glycine max* | Late Yangshao/Dahecun | 2.9 | 2.5 |  |
| Charred *Glycine max* | Late Yangshao/Dahecun | 2.8 | 2.1 |  |
| Charred *Glycine max* | Late Yangshao/Dahecun | 3.1 | 2.7 |  |
| Charred *Glycine max* | Late Yangshao/Dahecun | 2.5 | 2.3 |  |
| Charred *Glycine max* | Late Yangshao/Dahecun | 3.2 | 2.0 | 1.7 |
| Charred *Glycine max* | Late Yangshao/Dahecun | 3.3 | 2.3 | 1.9 |
| Charred *Glycine max* | Late Yangshao/Dahecun | 3.3 | 2.5 | 2.2 |
| Charred *Glycine max* | Late Yangshao/Dahecun | 3.2 | 2.4 | 1.8 |
| Charred *Glycine max* | Late Yangshao/Dahecun | 3.1 | 2.1 | 1.9 |
| Charred *Glycine max* | Late Yangshao/Dahecun | 3.4 | 2.6 | 1.9 |
| Charred *Glycine max* | Late Yangshao/Dahecun | 3.6 | 2.4 | 2.4 |
| Charred *Glycine max* | Late Yangshao/Dahecun | 3.0 | 2.2 | 2.1 |
| Charred *Glycine max* | Late Yangshao/Dahecun | 3.7 | 2.7 | 2.4 |
| Charred *Glycine max* | Late Yangshao/Dahecun | 3.2 | 2.6 | 2.3 |
| Charred *Glycine max* | Late Yangshao/Dahecun | 3.4 | 2.7 | 2.0 |
| Charred *Glycine max* | Late Yangshao/Dahecun | 4.2 | 2.3 | 2.5 |
| Charred *Glycine max* | Late Yangshao/Dahecun | 3.7 | 2.4 | 2.0 |
| Charred *Glycine max* | Late Yangshao/Dahecun | 3.3 | 2.1 | 2.0 |
| Charred *Glycine max* | Late Yangshao/Dahecun | 3.5 | 3.0 | 2.3 |
| Charred *Glycine max* | Late Yangshao/Dahecun | 3.2 | 2.8 | 2.4 |
| Charred *Glycine max* | Late Yangshao/Dahecun | 3.0 | 2.0 | 1.8 |
| Charred *Glycine max* | Late Yangshao/Dahecun | 3.5 | 2.7 | 2.2 |
| Charred *Glycine max* | Late Yangshao/Dahecun | 2.7 | 1.9 | 1.8 |
| Charred *Glycine max* | Late Yangshao/Dahecun | 3.1 | 2.6 | 2.6 |
| Charred *Glycine max* | Middle Jomon/Shimoyakebe | 6.0 | 4.1 | 2.9 |
| Charred *Glycine max* | Middle Jomon/Shimoyakebe | 7.4 | 4.4 | 3.0 |
| Charred *Glycine max* | Middle Jomon/Shimoyakebe | 7.6 | 4.7 | 3.9 |
| Charred *Glycine max* | Middle Jomon/Shimoyakebe | 8.2 | 5.3 | 3.1 |
| Charred *Glycine max* | Middle Jomon/Shimoyakebe | 7.3 | 4.1 | 3.1 |
| Charred *Glycine max* | Middle Jomon/Shimoyakebe | 7.6 | 4.4 | 3.3 |
| Charred *Glycine max* | Middle Jomon/Shimoyakebe | 5.9 | 3.8 | 2.9 |
| Charred *Glycine max* | Middle Jomon/Shimoyakebe | 6.3 | 3.7 | 2.7 |
| Charred *Glycine max* | Middle Jomon/Shimoyakebe | 6.1 | 4.1 | 2.6 |
| Charred *Glycine max* | Middle Jomon/Shimoyakebe | 5.7 | 3.6 | 3.1 |
| Charred *Glycine max* | Middle Jomon/Shimoyakebe | 8.3 | 3.6 | 5.0 |
| Charred *Glycine max* | Middle Jomon/Shimoyakebe | 6.7 | 2.8 | 3.6 |
| Charred *Glycine max* | Middle Jomon/Shimoyakebe | 7.2 | 4.1 | 3.3 |
| Charred *Glycine max* | Late Chulmun/Pyeonggeodong | 3.0 | 2.5 | 2.0 |
| Charred *Glycine max* | Late Chulmun/Pyeonggeodong | 2.7 | 1.9 | 1.6 |
| Charred *Glycine max* | Late Chulmun/Pyeonggeodong | 3.4 | 2.3 | 1.8 |
| Charred *Glycine max* | Late Chulmun/Pyeonggeodong | 3.9 | 2.5 | 2.1 |
| Charred *Glycine max* | Late Chulmun/Pyeonggeodong | 3.3 | 2.0 | 1.8 |
| Charred *Glycine max* | Late Chulmun/Pyeonggeodong | 3.2 | 1.8 | 1.1 |
| Charred *Glycine max* | Late Chulmun/Pyeonggeodong | 3.2 | 2.1 | 1.8 |
| Charred *Glycine max* | Late Chulmun/Pyeonggeodong | 3.5 | 3.1 |  |
| Charred *Glycine max* | Late Chulmun/Pyeonggeodong | 3.0 | 2.1 | 1.9 |
| Charred *Glycine max* | Late Chulmun/Pyeonggeodong | 3.0 | 2.3 | 1.9 |
| Charred *Glycine max* | Late Chulmun/Pyeonggeodong | 3.5 | 1.8 | 1.4 |
| Charred *Glycine max* | Late Chulmun/Pyeonggeodong | 3.4 | 2.0 | 2.0 |
| Charred *Glycine max* | Late Chulmun/Pyeonggeodong | 4.0 | 2.4 | 2.0 |
| Charred *Glycine max* | Late Chulmun/Pyeonggeodong | 3.1 | 1.9 | 1.3 |
| Charred *Glycine max* | Late Chulmun/Pyeonggeodong | 3.3 | 2.2 | 2.2 |
| Charred *Glycine max* | Late Chulmun/Pyeonggeodong | 2.9 | 2.4 |  |
| Charred *Glycine max* | Late Chulmun/Pyeonggeodong | 2.6 | 2.5 | 2.2 |
| Charred *Glycine max* | Late Chulmun/Pyeonggeodong | 3.0 | 2.5 |  |
| Charred *Glycine max* | Late Chulmun/Pyeonggeodong | 3.1 | 2.8 | 2.1 |
| Charred *Glycine max* | Late Longshan/Huizui | 3.8 | 3.0 |  |
| Charred *Glycine max* | Late Longshan/Huizui | 3.8 | 3.1 |  |
| Charred *Glycine max* | Late Longshan/Huizui | 1.7 | 0.9 |  |
| Charred *Glycine max* | Late Longshan/Huizui | 1.3 | 0.9 |  |
| Charred *Glycine max* | Late Longshan/Huizui | 3.9 | 2.5 |  |
| Charred *Glycine max* | Late Longshan/Huizui | 3.7 | 2.2 |  |
| Charred *Glycine max* | Late Longshan/Huizui | 4.4 | 2.3 |  |
| Charred *Glycine max* | Late Longshan/Huizui | 4.1 | 3.1 |  |
| Charred *Glycine max* | Late Longshan/Huizui | 1.7 | 1.0 |  |
| Charred *Glycine max* | Late Longshan/Huizui | 1.4 | 0.8 |  |
| Charred *Glycine max* | Late Longshan/Huizui | 4.8 | 3.3 |  |
| Charred *Glycine max* | Late Longshan/Jianxicun | 2.9 | 1.8 | 1.5 |
| Charred *Glycine max* | Late Longshan/Jianxicun | 2.5 | 1.5 | 1.4 |
| Charred *Glycine max* | Late Longshan/Huizui | 3.6 | 1.9 | 1.9 |
| Charred *Glycine max* | Late Longshan/Huizui | 4.6 | 2.7 |  |
| Charred *Glycine max* | Late Longshan/Huizui | 4.3 | 2.8 |  |
| Charred *Glycine max* | Late Longshan/Liangchengzhen | 4.6 | 2.4 | 1.7 |
| Charred *Glycine max* | Late Longshan/Liangchengzhen | 6.1 | 3.4 | 2.1 |
| Charred *Glycine max* | Late Longshan/Liangchengzhen | 4.2 | 2.7 | 1.6 |
| Charred *Glycine max* | Late Longshan/Liangchengzhen | 2.9 | 1.9 | 1.7 |
| Charred *Glycine max* | Late Longshan/Liangchengzhen | 2.5 | 1.5 | 1.4 |
| Charred *Glycine max* | Late Longshan/Liangchengzhen | 2.5 | 1.6 |  |
| Charred *Glycine max* | Late Longshan/Liangchengzhen | 4.6 | 3.0 |  |
| Charred *Glycine max* | Late Longshan/Liangchengzhen | 3.6 | 2.3 | 1.7 |
| Charred *Glycine max* | Late Longshan/Liangchengzhen | 2.3 | 1.3 | 1.0 |
| Charred *Glycine max* | Late Longshan/Liangchengzhen | 2.9 | 1.6 | 1.4 |
| Charred *Glycine max* | Late Longshan/Liangchengzhen | 3.1 | 1.9 | 1.9 |
| Charred *Glycine max* | Longshan/Xijingcheng | 4.7 | 2.9 |  |
| Charred *Glycine max* | Longshan/Xijingcheng | 4.3 | 2.5 |  |
| Charred *Glycine max* | Longshan/Shantaisi | 3.6 | 2.4 | 2.2 |
| Charred *Glycine max* | Longshan/Shantaisi | 2.4 | 1.4 | 1.3 |
| Charred *Glycine max* | Longshan/Shantaisi | 2.5 | 1.4 | 1.4 |
| Charred *Glycine max* | Longshan/Shantaisi | 2.4 | 1.5 | 1.5 |
| Charred *Glycine max* | Longshan/Shantaisi | 2.5 | 1.4 | 1.4 |
| Charred *Glycine max* | Longshan/Shantaisi | 2.6 | 1.3 | 1.2 |
| Charred *Glycine max* | Longshan/Shantaisi | 2.6 | 1.6 | 1.6 |
| Charred *Glycine max* | Longshan/Shantaisi | 3.7 | 3.2 | 2.3 |
| Charred *Glycine max* | Longshan/Shantaisi | 3.2 | 2.2 | 1.8 |
| Charred *Glycine max* | Longshan/Shantaisi | 3.9 | 2.6 | 1.8 |
| Charred *Glycine max* | Longshan/Shantaisi | 2.6 | 2.4 | 2.2 |
| Charred *Glycine max* | Longshan/Shantaisi | 3.1 | 1.9 | 1.7 |
| Charred *Glycine max* | Longshan/Shantaisi | 2.6 | 1.9 | 2.1 |
| Charred *Glycine max* | Longshan/Shantaisi | 2.5 | 2.1 | 1.6 |
| Charred *Glycine max* | Longshan/Shantaisi | 3.2 | 2.0 | 1.7 |
| Charred *Glycine max* | Longshan/Shantaisi | 2.6 | 2.0 | 1.4 |
| Charred *Glycine max* | Longshan/Shantaisi | 2.8 | 2.2 |  |
| Charred *Glycine max* | Longshan/Shantaisi | 2.2 | 1.4 |  |
| Charred *Glycine max* | Longshan/Shantaisi | 2.7 | 1.6 | 1.7 |
| Charred *Glycine max* | Longshan/Shantaisi | 2.4 | 1.6 | 1.0 |
| Charred *Glycine max* | Longshan/Shantaisi | 2.3 | 1.8 | 1.4 |
| Charred *Glycine max* | Longshan/Shantaisi | 2.6 | 1.5 | 1.9 |
| Charred *Glycine max* | Longshan/Shantaisi | 2.5 | 1.6 | 1.6 |
| Charred *Glycine max* | Longshan/Shantaisi | 2.1 | 1.7 | 1.4 |
| Charred *Glycine max* | Longshan/Shantaisi | 2.3 | 1.7 | 1.5 |
| Charred *Glycine max* | Longshan/Shantaisi | 2.4 | 1.5 | 1.4 |
| Charred *Glycine max* | Longshan/Shantaisi | 2.3 | 1.5 | 1.2 |
| Charred *Glycine max* | Longshan/Shantaisi | 2.6 | 1.6 | 1.4 |
| Charred *Glycine max* | Longshan/Shantaisi | 2.3 | 1.4 | 1.5 |
| Charred *Glycine max* | Longshan/Shantaisi | 2.8 | 1.9 | 1.4 |
| Charred *Glycine max* | Longshan/Shantaisi | 2.5 | 2.0 | 1.7 |
| Charred *Glycine max* | Longshan/Shantaisi | 2.3 | 2.1 | 1.4 |
| Charred *Glycine max* | Longshan/Shantaisi | 1.9 | 1.6 | 1.0 |
| Charred *Glycine max* | Longshan/Shantaisi | 2.3 | 1.7 | 1.4 |
| Charred *Glycine max* | Longshan/Zhouyuan | 6.2 | 4.3 | 2.6 |
| Charred *Glycine max* | Longshan/Zhouyuan | 5.4 | 4.3 | 2.7 |
| Charred *Glycine max* | Longshan/Zhouyuan | 5.7 | 4.3 | 2.7 |
| Charred *Glycine max* | Longshan/Zhouyuan | 5.6 | 3.6 | 3.2 |
| Charred *Glycine max* | Longshan/Zhouyuan | 4.2 | 2.6 | 2.2 |
| Charred *Glycine max* | Longshan/Zhouyuan | 4.4 | 3.3 | 2.3 |
| Charred *Glycine max* | Longshan/Zhouyuan | 5.1 | 2.5 | 2.3 |
| Charred *Glycine max* | Longshan/Zhouyuan | 4.6 | 3.6 | 3.1 |
| Charred *Glycine max* | Longshan/Zhouyuan | 4.0 | 2.9 | 1.9 |
| Charred *Glycine max* | Longshan/Zhouyuan | 5.0 | 3.8 | 2.5 |
| Charred *Glycine max* | Longshan/Zhouyuan | 4.3 | 3.1 | 2.5 |
| Charred *Glycine max* | Longshan/Zhouyuan | 4.0 | 2.3 | 1.6 |
| Charred *Glycine max* | Longshan/Zhouyuan | 4.7 | 2.9 | 2.3 |
| Charred *Glycine max* | Longshan/Zhouyuan | 4.5 | 3.4 | 2.5 |
| Charred *Glycine max* | Longshan/Zhouyuan | 4.9 | 3.5 | 2.9 |
| Charred *Glycine max* | Longshan/Zhouyuan | 4.2 | 3.2 | 2.4 |
| Charred *Glycine max* | Longshan/Zhouyuan | 4.8 | 3.9 | 3.2 |
| Charred *Glycine max* | Longshan/Zhouyuan | 5.5 | 4.5 | 2.9 |
| Charred *Glycine max* | Longshan/Zhouyuan | 4.3 | 2.7 | 2.3 |
| Charred *Glycine max* | Longshan/Zhouyuan | 4.0 | 2.5 | 2.2 |
| Charred *Glycine max* | Longshan/Wangchenggang | 4.8 | 2.9 | 1.9 |
| Charred *Glycine max* | Longshan/Wangchenggang | 4.7 | 2.7 | 2.1 |
| Charred *Glycine max* | Longshan/Wangchenggang | 4.3 | 2.9 | 2.3 |
| Charred *Glycine max* | Longshan/Wangchenggang | 4.7 | 2.8 | 2.1 |
| Charred *Glycine max* | Longshan/Wangchenggang | 4.9 | 3.7 | 2.6 |
| Charred *Glycine max* | Longshan/Wangchenggang | 5.0 | 2.9 | 2.9 |
| Charred *Glycine max* | Longshan/Wangchenggang | 4.9 | 4.0 | 2.6 |
| Charred *Glycine max* | Longshan/Wangchenggang | 4.6 | 3.4 | 2.7 |
| Charred *Glycine max* | Longshan/Wangchenggang | 5.1 | 3.3 | 2.5 |
| Charred *Glycine max* | Longshan/Wangchenggang | 4.7 | 3.9 | 2.6 |
| Charred *Glycine max* | Longshan/Wangchenggang | 4.4 | 3.0 | 2.5 |
| Charred *Glycine max* | Longshan/Wangchenggang | 4.6 | 3.7 | 2.6 |
| Charred *Glycine max* | Longshan/Wangchenggang | 4.6 | 3.2 | 2.0 |
| Charred *Glycine max* | Longshan/Wangchenggang | 4.6 | 3.4 | 2.4 |
| Charred *Glycine max* | Longshan/Wangchenggang | 4.9 | 3.5 | 3.0 |
| Charred *Glycine max* | Longshan/Wangchenggang | 3.9 | 2.9 | 2.5 |
| Charred *Glycine max* | Longshan/Wangchenggang | 4.1 | 3.1 | 2.4 |
| Charred *Glycine max* | Longshan/Wangchenggang | 5.1 | 3.4 | 2.7 |
| Charred *Glycine max* | Longshan/Wangchenggang | 4.5 | 3.3 | 2.8 |
| Charred *Glycine max* | Longshan/Wangchenggang | 4.5 | 4.0 | 2.8 |
| Charred *Glycine max* | Erlitou/Huizui | 5.3 | 3.1 |  |
| Charred *Glycine max* | Erlitou/Huizui | 5.3 | 2.8 |  |
| Charred *Glycine max* | Erlitou/Huizui | 3.8 | 2.2 |  |
| Charred *Glycine max* | Erlitou/Huizui | 3.4 | 2.3 |  |
| Charred *Glycine max* | Erlitou/Huizui | 4.6 | 2.6 | 1.7 |
| Charred *Glycine max* | Erlitou/Huizui | 3.0 | 2.4 |  |
| Charred *Glycine max* | Erlitou/Huizui | 4.9 | 3.5 |  |
| Charred *Glycine max* | Erlitou/Huizui | 3.4 | 2.1 |  |
| Charred *Glycine max* | Erlitou/Huizui | 3.4 | 2.2 |  |
| Charred *Glycine max* | Erlitou/Huizui | 5.4 | 3.5 | 3.3 |
| Charred *Glycine max* | Erlitou/Huizui | 3.5 | 2.0 |  |
| Charred *Glycine max* | Erlitou/Huizui | 4.7 | 2.7 | 2.5 |
| Charred *Glycine max* | Erlitou/Huizui | 3.6 | 2.0 |  |
| Charred *Glycine max* | Erlitou/Huizui | 4.4 | 2.3 |  |
| Charred *Glycine max* | Erlitou/Shaochai | 5.0 | 3.5 |  |
| Charred *Glycine max* | Erlitou/Shinzai | 4.5 | 2.8 | 2.9 |
| Charred *Glycine max* | Erlitou/Tianposhuiku N | 4.7 | 2.9 | 2.4 |
| Charred *Glycine max* | Erlitou/Tianposhuiku N | 3.4 | 1.3 |  |
| Charred *Glycine max* | Shang/Daxingzhuan | 2.4 | 1.5 | 1.1 |
| Charred *Glycine max* | Shang/Daxingzhuan | 2.7 | 2.0 | 1.6 |
| Charred *Glycine max* | Shang/Daxingzhuan | 2.3 | 2.0 | 1.8 |
| Charred *Glycine max* | Shang/Daxingzhuan | 2.7 | 2.1 | 1.9 |
| Charred *Glycine max* | Shang/Daxingzhuan | 2.9 | 1.8 | 1.6 |
| Charred *Glycine max* | Shang/Daxingzhuan | 2.3 | 1.9 | 1.7 |
| Charred *Glycine max* | Shang/Daxingzhuan | 2.2 | 1.8 | 1.6 |
| Charred *Glycine max* | Shang/Daxingzhuan | 2.4 | 2.2 | 1.4 |
| Charred *Glycine max* | Shang/Daxingzhuan | 2.1 | 1.6 | 1.4 |
| Charred *Glycine max* | Shang/Daxingzhuan | 2.4 | 1.7 | 1.1 |
| Charred *Glycine max* | Shang/Daxingzhuan | 2.7 | 2.0 | 1.8 |
| Charred *Glycine max* | Shang/Daxingzhuan | 2.0 | 1.4 | 1.4 |
| Charred *Glycine max* | Shang/Daxingzhuan | 2.1 | 1.8 | 1.5 |
| Charred *Glycine max* | Shang/Daxingzhuan | 2.1 | 1.6 | 1.3 |
| Charred *Glycine max* | Shang/Daxingzhuan | 2.2 | 1.7 | 1.5 |
| Charred *Glycine max* | Shang/Daxingzhuan | 2.5 | 1.7 | 1.1 |
| Charred *Glycine max* | Shang/Daxingzhuan | 2.1 | 1.6 | 1.4 |
| Charred *Glycine max* | Shang/Daxingzhuan | 2.0 | 1.8 | 1.1 |
| Charred *Glycine max* | Shang/Daxingzhuan | 2.3 | 1.9 | 1.3 |
| Charred *Glycine max* | Shang/Daxingzhuan | 2.2 | 1.8 | 1.3 |
| Charred *Glycine max* | Shang/Daxingzhuan | 2.2 | 1.9 | 1.5 |
| Charred *Glycine max* | Shang/Daxingzhuan | 2.1 | 2.0 | 1.6 |
| Charred *Glycine max* | Shang/Daxingzhuan | 2.4 | 1.9 | 1.3 |
| Charred *Glycine max* | Shang/Daxingzhuan | 4.8 | 3.1 | 2.2 |
| Charred *Glycine max* | Shang/Daxingzhuan | 5.6 | 3.5 | 2.4 |
| Charred *Glycine max* | Shang/Daxingzhuan | 4.0 | 2.8 | 1.9 |
| Charred *Glycine max* | Shang/Daxingzhuan | 5.6 | 3.4 | 2.5 |
| Charred *Glycine max* | Shang/Daxingzhuan | 4.7 | 3.3 | 2.8 |
| Charred *Glycine max* | Shang/Wangchenggang | 4.4 | 3.0 | 2.5 |
| Charred *Glycine max* | Shang/Wangchenggang | 4.9 | 3.2 | 2.4 |
| Charred *Glycine max* | Shang/Wangchenggang | 6.4 | 4.3 | 2.9 |
| Charred *Glycine max* | Shang/Wangchenggang | 6.1 | 4.7 | 2.7 |
| Charred *Glycine max* | Shang/Wangchenggang | 4.0 | 3.2 | 2.7 |
| Charred *Glycine max* | Shang/Wangchenggang | 5.2 | 3.8 | 2.6 |
| Charred *Glycine max* | Proto-Zhou/Zhouyuan | 4.2 | 2.9 | 2.2 |
| Charred *Glycine max* | Proto-Zhou/Zhouyuan | 5.1 | 3.1 | 2.7 |
| Charred *Glycine max* | Proto-Zhou/Zhouyuan | 5.0 | 3.3 | 2.6 |
| Charred *Glycine max* | Proto-Zhou/Zhouyuan | 4.9 | 3.0 | 2.8 |
| Charred *Glycine max* | Proto-Zhou/Zhouyuan | 4.1 | 2.7 | 2.2 |
| Charred *Glycine max* | Proto-Zhou/Zhouyuan | 5.0 | 3.4 | 2.5 |
| Charred *Glycine max* | Proto-Zhou/Zhouyuan | 4.2 | 2.4 | 1.8 |
| Charred *Glycine max* | Proto-Zhou/Zhouyuan | 4.5 | 2.9 | 2.5 |
| Charred *Glycine max* | Proto-Zhou/Zhouyuan | 4.9 | 3.2 | 2.4 |
| Charred *Glycine max* | Early Mumun/Nam R | 7.0 | 4.2 |  |
| Charred *Glycine max* | Early Mumun/Nam R | 7.7 | 5.0 | 2.9 |
| Charred *Glycine max* | Early Mumun/Nam R | 7.0 | 3.8 |  |
| Charred *Glycine max* | Early Mumun/Nam R | 6.8 |  |  |
| Charred *Glycine max* | Early Mumun/Nam R | 5.0 | 3.5 | 2.5 |
| Charred *Glycine max* | Early Mumun/Nam R | 5.2 | 3.3 |  |
| Charred *Glycine max* | Early Mumun/Nam R | 4.7 | 3.3 |  |
| Charred *Glycine max* | Early Mumun/Nam R | 4.0 |  |  |
| Charred *Glycine max* | Early Mumun/Nam R | 6.3 | 6.1 | 2.0 |
| Charred *Glycine max* | Middle Mumun/Daundong | 6.8 | 5.2 | 4.4 |
| Charred *Glycine max* | Middle Mumun/Daundong | 8.2 | 5.1 | 5.6 |
| Charred *Glycine max* | Middle Mumun/Daundong | 7.4 | 4.9 | 4.2 |
| Charred *Glycine max* | Middle Mumun/Daundong | 6.3 | 4.7 | 3.0 |
| Charred *Glycine max* | Middle Mumun/Daundong | 7.1 | 5.0 | 4.6 |
| Charred *Glycine max* | Middle Mumun/Daundong | 7.2 | 5.8 | 4.9 |
| Charred *Glycine max* | Middle Mumun/Daundong | 7.1 | 4.3 | 3.8 |
| Charred *Glycine max* | Middle Mumun/Daundong | 7.2 | 4.9 | 4.5 |
| Charred *Glycine max* | Middle Mumun/Daundong | 7.3 | 4.6 | 4.0 |
| Charred *Glycine max* | Middle Mumun/Daundong | 6.8 | 4.1 | 5.8 |
| Charred *Glycine max* | Middle Mumun/Daundong | 5.8 | 4.8 | 4.5 |
| Charred *Glycine max* | Middle Mumun/Daundong | 6.3 | 4.4 | 4.5 |
| Charred *Glycine max* | Middle Mumun/Daundong | 7.0 | 4.5 | 3.8 |
| Charred *Glycine max* | Middle Mumun/Daundong | 7.0 | 5.0 | 3.8 |
| Charred *Glycine max* | Middle Mumun/Daundong | 6.8 | 5.0 | 3.3 |
| Charred *Glycine max* | Middle Mumun/Daundong | 7.0 | 4.8 |  |
| Charred *Glycine max* | Middle Mumun/Daundong | 7.3 | 5.3 |  |
| Charred *Glycine max* | Middle Mumun/Daundong | 7.0 |  |  |
| Charred *Glycine max* | Middle Mumun/Daundong | 7.3 |  |  |
| Charred *Glycine max* | Middle Mumun/Daundong | 7.7 | 5.6 | 5.0 |
| Charred *Glycine max* | Middle Mumun/Daundong | 7.0 |  |  |
| Charred *Glycine max* | Middle Mumun/Daundong | 5.8 | 4.0 |  |
| Charred *Glycine max* | Middle Mumun/Daundong | 6.0 | 4.5 | 4.3 |
| Charred *Glycine max* | Middle Mumun/Daundong | 5.9 | 4.5 | 4.3 |
| Charred *Glycine max* | Middle Mumun/Daundong | 7.0 | 5.0 |  |
| Charred *Glycine max* | Middle Mumun/Daundong | 6.9 | 5.1 |  |
| Charred *Glycine max* | Middle Mumun/Daundong | 7.4 | 4.7 |  |
| Charred *Glycine max* | Middle Mumun/Daundong | 7.1 | 5.3 | 4.2 |
| Charred *Glycine max* | Middle Mumun/Daundong | 8.0 |  |  |
| Charred *Glycine max* | Middle Mumun/Daundong | 6.9 |  |  |
| Charred *Glycine max* | Middle Mumun/Daundong | 8.0 |  |  |
| Charred *Glycine max* | Middle Mumun/Daundong | 7.0 |  |  |
| Charred *Glycine max* | Middle Mumun/Daundong | 8.1 |  |  |
| Charred *Glycine max* | Middle Mumun/Daundong | 6.0 |  |  |
| Charred *Glycine max* | Middle Mumun/Daundong | 7.0 |  |  |
| Charred *Glycine max* | Middle Mumun/Daundong | 7.2 | 4.5 | 4.0 |
| Charred *Glycine max* | Middle Mumun/Daundong | 7.0 | 4.8 | 4.5 |
| Charred *Glycine max* | Middle Mumun/Daundong | 7.3 | 4.8 |  |
| Charred *Glycine max* | Middle Mumun/Daundong | 6.8 | 5.0 | 4.5 |
| Charred *Glycine max* | Middle Mumun/Daundong | 6.8 | 4.5 | 4.3 |
| Charred *Glycine max* | Middle Mumun/Daundong | 7.0 | 4.7 | 4.8 |
| Charred *Glycine max* | Middle Mumun/Daundong | 7.1 | 5.0 | 4.7 |
| Charred *Glycine max* | Middle Mumun/Daundong | 6.7 | 4.9 | 3.7 |
| Charred *Glycine max* | Middle Mumun/Daundong | 7.2 | 5.5 | 4.5 |
| Charred *Glycine max* | Middle Mumun/Daundong | 7.0 | 4.3 |  |
| Charred *Glycine max* | Middle Mumun/Daundong | 7.4 | 4.8 | 4.0 |
| Charred *Glycine max* | Middle Mumun/Daundong | 7.2 | 6.0 | 4.5 |
| Charred *Glycine max* | Middle Mumun/Daundong | 5.5 | 4.7 |  |
| Charred *Glycine max* | Middle Mumun/Daundong | 6.1 | 4.7 |  |
| Charred *Glycine max* | Middle Mumun/Daundong | 7.7 | 5.8 | 4.7 |
| Charred *Glycine max* | Middle Mumun/Daundong | 6.7 | 5.1 | 4.1 |
| Charred *Glycine max* | Middle Mumun/Daundong | 3.8 | 2.9 | 2.7 |
| Charred *Glycine max* | Middle Mumun/Daundong | 7.2 | 5.8 | 5.5 |
| Charred *Glycine max* | Middle Mumun/Daundong | 7.0 | 5.8 | 5.1 |
| Charred *Glycine max* | Middle Mumun/Daundong | 6.7 | 5.4 |  |
| Charred *Glycine max* | Middle Mumun/Daundong | 7.2 | 5.6 | 5.0 |
| Charred *Glycine max* | Middle Mumun/Daundong | 7.7 | 5.0 |  |
| Charred *Glycine max* | Middle Mumun/Daundong | 7.4 | 5.5 | 5.0 |
| Charred *Glycine max* | Middle Mumun/Daundong | 7.0 | 5.5 | 5.0 |
| Charred *Glycine max* | Middle Mumun/Daundong | 6.1 |  | 3.8 |
| Charred *Glycine max* | Middle Mumun/Daundong | 7.0 | 5.7 | 5.0 |
| Charred *Glycine max* | Middle Mumun/Daundong | 7.0 | 4.9 | 4.4 |
| Charred *Glycine max* | Middle Mumun/Daundong | 8.4 | 6.1 | 5.0 |
| Charred *Glycine max* | Middle Mumun/Daundong | 7.7 | 5.2 | 5.1 |
| Charred *Glycine max* | Middle Mumun/Daundong | 6.7 | 5.2 | 4.9 |
| Charred *Glycine max* | Middle Mumun/Daundong | 9.0 | 6.5 | 5.1 |
| Charred *Glycine max* | Middle Mumun/Daundong | 9.3 | 5.7 | 5.5 |
| Charred *Glycine max* | Middle Mumun/Daundong | 9.5 | 6.4 | 5.4 |
| Charred *Glycine max* | Middle Mumun/Daundong | 7.8 | 5.5 | 5.0 |
| Charred *Glycine max* | Middle Mumun/Daundong | 8.5 | 5.5 | 5.4 |
| Charred *Glycine max* | Middle Mumun/Daundong | 6.3 | 5.8 | 4.5 |
| Charred *Glycine max* | Middle Mumun/Daundong | 9.0 | 6.1 |  |
| Charred *Glycine max* | Middle Mumun/Daundong | 8.2 | 5.4 | 5.1 |
| Charred *Glycine max* | Middle Mumun/Daundong | 7.6 | 5.2 |  |
| Charred *Glycine max* | Middle Mumun/Daundong | 7.0 | 5.2 | 4.7 |
| Charred *Glycine max* | Middle Mumun/Daundong | 7.4 | 5.7 | 5.0 |
| Charred *Glycine max* | Middle Mumun/Daundong | 9.1 | 6.3 | 5.8 |
| Charred *Glycine max* | Middle Mumun/Daundong | 7.7 | 5.0 | 4.8 |
| Charred *Glycine max* | Middle Mumun/Daundong | 7.3 | 5.2 | 5.0 |
| Charred *Glycine max* | Middle Mumun/Daundong | 7.0 | 5.4 |  |
| Charred *Glycine max* | Middle Mumun/Daundong | 7.2 | 4.8 | 4.6 |
| Charred *Glycine max* | Middle Mumun/Daundong | 7.4 | 5.2 | 5.0 |
| Charred *Glycine max* | Middle Mumun/Daundong | 9.0 | 6.0 | 5.8 |
| Charred *Glycine max* | Middle Mumun/Daundong | 8.5 | 5.6 | 5.2 |
| Charred *Glycine max* | Middle Mumun/Daundong | 5.3 | 4.3 |  |
| Charred *Glycine max* | Middle Mumun/Daundong | 7.6 | 5.5 | 5.3 |
| Charred *Glycine max* | Middle Mumun/Daundong | 7.4 | 5.8 |  |
| Charred *Glycine max* | Middle Mumun/Daundong | 7.2 |  | 4.0 |
| Charred *Glycine max* | Middle Mumun/Daundong | 8.1 | 6.3 |  |
| Charred *Glycine max* | Middle Mumun/Daundong | 7.2 | 5.7 |  |
| Charred *Glycine max* | Middle Mumun/Daundong | 7.6 | 5.0 | 4.9 |
| Charred *Glycine max* | Middle Mumun/Daundong | 7.2 | 5.3 |  |
| Charred *Glycine max* | Middle Mumun/Daundong | 7.5 | 5.5 | 5.1 |
| Charred *Glycine max* | Middle Mumun/Daundong | 7.1 | 5.0 | 4.8 |
| Charred *Glycine max* | Middle Mumun/Daundong | 8.2 | 5.6 | 5.7 |
| Charred *Glycine max* | Middle Mumun/Daundong | 6.5 | 5.0 | 4.7 |
| Charred *Glycine max* | Middle Mumun/Daundong | 7.7 |  | 4.8 |
| Charred *Glycine max* | Middle Mumun/Daundong | 7.2 | 5.5 |  |
| Charred *Glycine max* | Middle Mumun/Daundong | 6.8 | 4.8 | 4.8 |
| Charred *Glycine max* | Middle Mumun/Daundong | 8.2 | 6.4 |  |
| Charred *Glycine max* | Middle Mumun/Daundong | 9.2 | 6.2 | 4.4 |
| Charred *Glycine max* | Middle Mumun/Daundong | 7.2 | 5.4 | 4.9 |
| Charred *Glycine max* | Middle Mumun/Daundong | 7.7 | 5.8 | 5.1 |
| Charred *Glycine max* | Middle Mumun/Daundong | 8.3 | 5.7 | 5.5 |
| Charred *Glycine max* | Middle Mumun/Daundong | 8.4 | 7.0 | 5.5 |
| Charred *Glycine max* | Middle Mumun/Daundong | 7.0 | 5.3 | 4.6 |
| Charred *Glycine max* | Middle Mumun/Daundong | 7.5 | 5.7 | 5.4 |
| Charred *Glycine max* | Middle Mumun/Daundong | 7.0 | 5.7 | 4.5 |
| Charred *Glycine max* | Middle Mumun/Daundong | 5.7 | 3.8 | 3.5 |
| Charred *Glycine max* | Middle Mumun/Daundong | 6.7 | 5.3 | 5.0 |
| Charred *Glycine max* | Middle Mumun/Daundong | 7.0 | 5.3 | 5.1 |
| Charred *Glycine max* | Middle Mumun/Daundong | 7.2 | 4.8 | 4.8 |
| Charred *Glycine max* | Middle Mumun/Daundong | 6.8 | 4.8 | 4.5 |
| Charred *Glycine max* | Middle Mumun/Daundong | 7.6 | 5.5 |  |
| Charred *Glycine max* | Middle Mumun/Daundong | 8.0 | 6.1 |  |
| Charred *Glycine max* | Middle Mumun/Daundong | 6.1 | 4.7 | 4.0 |
| Charred *Glycine max* | Middle Mumun/Daundong | 7.2 | 5.5 | 4.9 |
| Charred *Glycine max* | Middle Mumun/Daundong | 7.8 | 5.4 |  |
| Charred *Glycine max* | Middle Mumun/Daundong | 9.4 | 5.5 |  |
| Charred *Glycine max* | Middle Mumun/Daundong | 8.9 | 5.7 | 5.4 |
| Charred *Glycine max* | Middle Mumun/Daundong | 7.8 | 5.5 | 5.0 |
| Charred *Glycine max* | Middle Mumun/Daundong | 7.3 | 6.0 | 3.6 |
| Charred *Glycine max* | Middle Mumun/Daundong | 7.8 | 6.4 | 6.4 |
| Charred *Glycine max* | Middle Mumun/Daundong | 7.6 | 6.2 | 4.5 |
| Charred *Glycine max* | Middle Mumun/Daundong | 6.5 | 5.0 | 3.9 |
| Charred *Glycine max* | Middle Mumun/Daundong | 8.2 | 5.3 |  |
| Charred *Glycine max* | Middle Mumun/Daundong | 8.1 | 5.5 | 4.5 |
| Charred *Glycine max* | Middle Mumun/Daundong | 7.9 | 5.3 |  |
| Charred *Glycine max* | Middle Mumun/Daundong | 7.0 | 5.7 | 5.0 |
| Charred *Glycine max* | Middle Mumun/Daundong | 9.0 | 6.0 | 5.2 |
| Charred *Glycine max* | Middle Mumun/Daundong | 7.5 | 5.0 | 4.5 |
| Charred *Glycine max* | Middle Mumun/Daundong | 7.7 | 6.0 | 5.2 |
| Charred *Glycine max* | Middle Mumun/Daundong | 8.1 | 5.8 |  |
| Charred *Glycine max* | Middle Mumun/Daundong | 8.7 | 6.7 | 6.3 |
| Charred *Glycine max* | Middle Mumun/Daundong | 6.2 | 4.7 | 4.4 |
| Charred *Glycine max* | Middle Mumun/Daundong | 7.1 | 5.4 | 5.0 |
| Charred *Glycine max* | Middle Mumun/Daundong | 7.5 | 5.5 | 5.1 |
| Charred *Glycine max* | Middle Mumun/Daundong | 6.5 | 4.7 |  |
| Charred *Glycine max* | Middle Mumun/Daundong | 7.6 | 5.6 |  |
| Charred *Glycine max* | Middle Mumun/Daundong | 7.5 | 5.9 | 5.3 |
| Charred *Glycine max* | Middle Mumun/Daundong | 6.5 | 4.5 | 4.2 |
| Charred *Glycine max* | Middle Mumun/Daundong | 6.7 | 4.5 | 4.3 |
| Charred *Glycine max* | Middle Mumun/Daundong | 6.0 | 4.5 | 4.0 |
| Charred *Glycine max* | Middle Mumun/Daundong | 7.4 | 5.1 | 4.4 |
| Charred *Glycine max* | Middle Mumun/Daundong | 8.6 | 5.1 | 4.5 |
| Charred *Glycine max* | Middle Mumun/Daundong | 6.4 | 4.0 |  |
| Charred *Glycine max* | Middle Mumun/Daundong | 5.3 | 4.0 | 3.7 |
| Charred *Glycine max* | Middle Mumun/Daundong | 7.5 | 5.7 | 5.0 |
| Charred *Glycine max* | Middle Mumun/Daundong | 7.0 | 5.5 | 4.9 |
| Charred *Glycine max* | Middle Mumun/Daundong | 8.3 | 5.0 | 4.5 |
| Charred *Glycine max* | Middle Mumun/Daundong | 7.2 | 5.0 |  |
| Charred *Glycine max* | Middle Mumun/Daundong | 7.0 | 6.0 | 4.7 |
| Charred *Glycine max* | Middle Mumun/Daundong | 6.8 | 5.4 | 5.0 |
| Charred *Glycine max* | Middle Mumun/Daundong | 7.5 | 5.5 | 5.2 |
| Charred *Glycine max* | Middle Mumun/Daundong | 7.0 | 6.1 |  |
| Charred *Glycine max* | Middle Mumun/Daundong | 7.0 | 5.5 | 3.9 |
| Charred *Glycine max* | Middle Mumun/Daundong | 9.0 | 6.1 | 5.0 |
| Charred *Glycine max* | Middle Mumun/Daundong | 7.8 | 6.0 | 4.8 |
| Charred *Glycine max* | Middle Mumun/Daundong | 9.3 | 5.3 | 4.5 |
| Charred *Glycine max* | Middle Mumun/Daundong | 8.5 | 6.1 |  |
| Charred *Glycine max* | Middle Mumun/Daundong | 8.5 | 6.3 |  |
| Charred *Glycine max* | Middle Mumun/Daundong | 9.0 | 6.0 | 4.5 |
| Charred *Glycine max* | Middle Mumun/Daundong | 7.5 | 5.5 |  |
| Charred *Glycine max* | Middle Mumun/Daundong | 7.5 | 5.0 |  |
| Charred *Glycine max* | Middle Mumun/Daundong | 8.4 | 5.5 | 5.4 |
| Charred *Glycine max* | Middle Mumun/Daundong | 8.1 | 5.7 |  |
| Charred *Glycine max* | Middle Mumun/Daundong | 7.9 | 5.5 | 5.1 |
| Charred *Glycine max* | Middle Mumun/Daundong | 7.8 | 5.0 |  |
| Charred *Glycine max* | Middle Mumun/Daundong | 9.3 | 5.6 | 5.1 |
| Charred *Glycine max* | Middle Mumun/Daundong | 9.0 | 6.4 | 6.2 |
| Charred *Glycine max* | Middle Mumun/Daundong | 9.0 | 6.4 | 6.2 |
| Charred *Glycine max* | Middle Mumun/Daundong | 6.0 | 5.0 | 4.8 |
| Charred *Glycine max* | Middle Mumun/Daundong | 8.8 | 6.0 |  |
| Charred *Glycine max* | Middle Mumun/Daundong | 6.6 | 5.0 | 4.8 |
| Charred *Glycine max* | Middle Mumun/Daundong | 8.7 | 7.4 | 5.3 |
| Charred *Glycine max* | Middle Mumun/Daundong | 8.7 | 6.0 | 5.5 |
| Charred *Glycine max* | Middle Mumun/Daundong | 7.7 | 4.5 |  |
| Charred *Glycine max* | Middle Mumun/Daundong | 7.6 | 6.0 |  |
| Charred *Glycine max* | Middle Mumun/Daundong | 7.7 | 4.8 |  |
| Charred *Glycine max* | Middle Mumun/Daundong | 7.2 |  | 5.3 |
| Charred *Glycine max* | Middle Mumun/Daundong | 7.6 | 5.5 |  |
| Charred *Glycine max* | Middle Mumun/Daundong | 7.1 | 5.7 | 5.0 |
| Charred *Glycine max* | Middle Mumun/Daundong | 6.9 | 5.3 |  |
| Charred *Glycine max* | Middle Mumun/Daundong | 7.6 | 5.5 | 5.3 |
| Charred *Glycine max* | Middle Mumun/Daundong | 8.1 | 6.5 | 5.4 |
| Charred *Glycine max* | Middle Mumun/Daundong | 7.6 | 4.8 | 4.3 |
| Charred *Glycine max* | Middle Mumun/Daundong | 6.5 | 5.0 | 4.8 |
| Charred *Glycine max* | Middle Mumun/Daundong | 7.5 | 4.5 | 4.0 |
| Charred *Glycine max* | Middle Mumun/Daundong | 9.5 | 6.0 | 5.3 |
| Charred *Glycine max* | Middle Mumun/Daundong | 7.2 | 6.0 | 5.2 |
| Charred *Glycine max* | Middle Mumun/Daundong | 8.0 | 6.3 | 6.2 |
| Charred *Glycine max* | Middle Mumun/Daundong | 9.0 | 5.8 | 5.6 |
| Charred *Glycine max* | Middle Mumun/Daundong | 7.3 |  | 4.9 |
| Charred *Glycine max* | Middle Mumun/Daundong | 8.5 | 5.8 | 5.2 |
| Charred *Glycine max* | Middle Mumun/Daundong | 7.1 | 5.0 | 4.8 |
| Charred *Glycine max* | Middle Mumun/Daundong | 7.3 | 5.0 |  |
| Charred *Glycine max* | Middle Mumun/Daundong | 7.4 | 5.8 | 5.2 |
| Charred *Glycine max* | Middle Mumun/Daundong | 8.0 | 5.5 | 5.5 |
| Charred *Glycine max* | Middle Mumun/Daundong | 8.2 | 6.0 |  |
| Charred *Glycine max* | Middle Mumun/Daundong | 7.8 | 6.2 | 5.7 |
| Charred *Glycine max* | Middle Mumun/Daundong | 6.9 | 5.2 |  |
| Charred *Glycine max* | Middle Mumun/Daundong | 6.2 | 4.7 | 3.3 |
| Charred *Glycine max* | Middle Mumun/Daundong | 6.5 | 4.8 | 4.3 |
| Charred *Glycine max* | Middle Mumun/Daundong | 8.3 | 6.0 | 5.0 |
| Charred *Glycine max* | Middle Mumun/Daundong | 9.2 | 6.2 |  |
| Charred *Glycine max* | Middle Mumun/Daundong | 8.0 | 5.7 | 6.9 |
| Charred *Glycine max* | Middle Mumun/Daundong | 8.0 | 5.6 | 5.4 |
| Charred *Glycine max* | Middle Mumun/Daundong | 6.5 | 4.5 | 4.1 |
| Charred *Glycine max* | Middle Mumun/Daundong | 7.1 | 5.6 | 5.6 |
| Charred *Glycine max* | Middle Mumun/Daundong | 6.6 | 4.7 | 4.6 |
| Charred *Glycine max* | Middle Mumun/Daundong | 7.8 | 5.6 |  |
| Charred *Glycine max* | Middle Mumun/Daundong | 9.0 | 5.4 |  |
| Charred *Glycine max* | Middle Mumun/Daundong | 8.3 | 5.5 | 4.2 |
| Charred *Glycine max* | Middle Mumun/Daundong | 8.6 | 5.5 |  |
| Charred *Glycine max* | Middle Mumun/Daundong | 8.5 | 6.5 | 5.5 |
| Charred *Glycine max* | Middle Mumun/Daundong | 8.5 | 6.6 | 6.1 |
| Charred *Glycine max* | Middle Mumun/Daundong | 7.3 | 6.0 | 5.8 |
| Charred *Glycine max* | Middle Mumun/Daundong | 9.9 | 6.8 |  |
| Charred *Glycine max* | Middle Mumun/Daundong | 7.0 | 5.8 |  |
| Charred *Glycine max* | Middle Mumun/Daundong | 8.0 | 5.7 | 5.0 |
| Charred *Glycine max* | Middle Mumun/Daundong | 9.5 | 6.0 | 5.8 |
| Charred *Glycine max* | Middle Mumun/Daundong | 7.8 | 5.2 |  |
| Charred *Glycine max* | Middle Mumun/Daundong | 6.9 | 5.0 | 5.0 |
| Charred *Glycine max* | Middle Mumun/Daundong | 6.9 | 5.5 |  |
| Charred *Glycine max* | Middle Mumun/Daundong | 8.0 | 6.0 | 5.8 |
| Charred *Glycine max* | Middle Mumun/Daundong | 7.2 | 5.5 | 5.5 |
| Charred *Glycine max* | Middle Mumun/Daundong | 7.6 | 5.6 | 5.0 |
| Charred *Glycine max* | Middle Mumun/Daundong | 8.7 | 5.5 | 5.0 |
| Charred *Glycine max* | Middle Mumun/Daundong | 7.5 | 5.3 |  |
| Charred *Glycine max* | Middle Mumun/Daundong | 7.5 | 4.8 | 4.7 |
| Charred *Glycine max* | Middle Mumun/Daundong | 8.0 | 5.6 | 5.6 |
| Charred *Glycine max* | Middle Mumun/Daundong | 5.7 | 4.7 | 4.0 |
| Charred *Glycine max* | Middle Mumun/Daundong | 8.0 | 5.6 |  |
| Charred *Glycine max* | Middle Mumun/Daundong | 7.6 | 5.0 | 4.7 |
| Charred *Glycine max* | Middle Mumun/Daundong | 7.0 | 5.4 | 5.4 |
| Charred *Glycine max* | Middle Mumun/Daundong | 6.1 | 4.9 |  |
| Charred *Glycine max* | Middle Mumun/Daundong | 6.1 | 3.8 |  |
| Charred *Glycine max* | Middle Mumun/Daundong | 7.9 | 5.4 | 5.0 |
| Charred *Glycine max* | Middle Mumun/Daundong | 5.7 | 4.5 | 3.5 |
| Charred *Glycine max* | Middle Mumun/Daundong | 9.0 | 6.1 | 6.0 |
| Charred *Glycine max* | Middle Mumun/Daundong | 7.3 | 5.0 |  |
| Charred *Glycine max* | Middle Mumun/Daundong | 8.7 | 6.0 |  |
| Charred *Glycine max* | Middle Mumun/Daundong | 5.8 | 4.6 | 4.3 |
| Charred *Glycine max* | Middle Mumun/Daundong | 5.5 | 4.3 |  |
| Charred *Glycine max* | Middle Mumun/Daundong | 6.5 | 5.5 | 5.2 |
| Charred *Glycine max* | Middle Mumun/Daundong | 8.0 | 5.8 | 5.5 |
| Charred *Glycine max* | Middle Mumun/Daundong | 7.8 | 5.0 |  |
| Charred *Glycine max* | Middle Mumun/Daundong | 8.0 | 5.7 | 5.5 |
| Charred *Glycine max* | Middle Mumun/Daundong | 8.1 | 5.7 | 4.5 |
| Charred *Glycine max* | Middle Mumun/Daundong | 7.5 | 4.9 |  |
| Charred *Glycine max* | Middle Mumun/Daundong | 8.3 | 6.0 |  |
| Charred *Glycine max* | Middle Mumun/Daundong | 7.1 | 5.2 |  |
| Charred *Glycine max* | Middle Mumun/Daundong | 6.5 | 4.9 | 4.8 |
| Charred *Glycine max* | Middle Mumun/Daundong | 7.9 | 5.0 |  |
| Charred *Glycine max* | Middle Mumun/Daundong | 8.2 | 6.0 |  |
| Charred *Glycine max* | Middle Mumun/Daundong | 6.7 | 5.1 | 4.9 |
| Charred *Glycine max* | Middle Mumun/Daundong | 8.0 | 6.0 | 5.7 |
| Charred *Glycine max* | Middle Mumun/Daundong | 8.3 | 6.0 |  |
| Charred *Glycine max* | Middle Mumun/Daundong | 8.1 | 5.5 |  |
| Charred *Glycine max* | Middle Mumun/Daundong | 7.5 | 5.5 |  |
| Charred *Glycine max* | Middle Mumun/Daundong | 6.9 | 4.7 |  |
| Charred *Glycine max* | Middle Mumun/Daundong | 6.8 | 4.4 |  |
| Charred *Glycine max* | Middle Mumun/Daundong | 9.5 | 6.5 | 5.5 |
| Charred *Glycine max* | Middle Mumun/Daundong | 6.5 | 4.7 |  |
| Charred *Glycine max* | Middle Mumun/Daundong | 6.6 | 4.7 | 3.8 |
| Charred *Glycine max* | Middle Mumun/Daundong | 6.7 | 4.4 | 4.0 |
| Charred *Glycine max* | Middle Mumun/Daundong | 6.9 | 5.0 | 5.0 |
| Charred *Glycine max* | Middle Mumun/Daundong | 7.0 | 5.2 |  |
| Charred *Glycine max* | Middle Mumun/Daundong | 6.5 | 4.5 |  |
| Charred *Glycine max* | Middle Mumun/Daundong | 6.2 | 4.2 | 4.0 |
| Charred *Glycine max* | Middle Mumun/Daundong | 7.1 | 5.2 | 4.0 |
| Charred *Glycine max* | Middle Mumun/Daundong | 5.5 | 4.3 | 4.3 |
| Charred *Glycine max* | Middle Mumun/Daundong | 6.5 | 4.5 | 4.5 |
| Charred *Glycine max* | Middle Mumun/Daundong | 7.7 | 5.9 | 5.4 |
| Charred *Glycine max* | Middle Mumun/Daundong | 7.0 | 4.5 | 4.2 |
| Charred *Glycine max* | Middle Mumun/Daundong | 8.0 | 6.6 |  |
| Charred *Glycine max* | Middle Mumun/Daundong | 7.2 | 5.2 | 5.2 |
| Charred *Glycine max* | Middle Mumun/Daundong | 7.4 | 5.0 | 5.0 |
| Charred *Glycine max* | Han/Xijingcheng | 5.5 | 4.3 |  |
| Charred *Glycine max* | Han/Xijingcheng | 5.3 | 3.8 |  |
| Charred *Glycine max* | Han/Xijingcheng | 5.1 | 4.0 |  |
| Charred *Glycine max* | Han/Xijingcheng | 5.1 | 4.4 |  |
| Charred *Glycine max* | Han/Xijingcheng | 5.2 | 3.8 |  |
| Charred *Glycine max* | Han/Xijingcheng | 6.3 | 3.9 |  |
| Charred *Glycine max* | Han/Xijingcheng | 6.5 | 4.4 |  |
| Charred *Glycine max* | Han/Xijingcheng | 5.2 | 3.8 |  |
| Charred *Glycine max* | Han/Xijingcheng | 4.9 | 4.3 |  |
| Charred *Glycine max* | Han/Xijingcheng | 5.1 | 4.5 |  |
| Charred *Glycine max* | Han/Xijingcheng | 4.9 | 4.0 |  |
| Charred *Glycine max* | Han/Xijingcheng | 5.0 | 4.3 |  |
| Charred *Glycine max* | Han/Xijingcheng | 6.4 | 3.7 |  |
| Charred *Glycine max* | Han/Xijingcheng | 5.2 | 4.1 |  |
| Charred *Glycine max* | Han/Xijingcheng | 5.0 | 4.1 |  |
| Charred *Glycine max* | Han/Xijingcheng | 4.6 | 3.8 |  |
| Charred *Glycine max* | Han/Xijingcheng | 5.7 | 3.8 |  |
| Charred *Glycine max* | Han/Xijingcheng | 4.9 | 4.0 |  |
| Charred *Glycine max* | Han/Xijingcheng | 5.4 | 4.7 |  |
| Charred *Glycine max* | Han/Xijingcheng | 5.5 | 4.7 |  |
| Charred *Glycine max* | Three Kingdom/Nam R | 8.3 | 5.5 | 2.2 |
| Charred *Glycine max* | Three Kingdom/Nam R | 8.0 | 5.0 |  |
| Charred *Glycine max* | Three Kingdom/Nam R | 6.0 |  |  |
| Charred *Glycine max* | Three Kingdom/Nam R | 7.0 |  |  |
| Charred *Glycine max* | Three Kingdom/Nam R | 6.9 | 4.7 |  |
| Charred *Glycine max* | Three Kingdom/Nam R | 6.8 | 4.2 | 2.1 |
| Charred *Glycine max* | Three Kingdom/Nam R | 6.4 | 4.0 |  |
| Charred *Glycine max* | Three Kingdom/Nam R | 6.3 | 4.2 | 2.1 |
| Charred *Glycine max* | Three Kingdom/Nam R | 7.0 | 3.7 |  |
| Charred *Glycine max* | Three Kingdom/Nam R | 6.7 | 3.8 |  |
| Charred *Glycine max* | Three Kingdom/Nam R | 6.3 | 4.3 |  |
| Charred *Glycine max* | Three Kingdom/Nam R | 6.1 | 3.5 |  |
| Charred *Glycine max* | Three Kingdom/Nam R | 7.4 | 4.0 | 3.0 |
| Charred *Glycine max* | Three Kingdom/Nam R | 6.6 | 3.6 | 3.0 |
| Charred *Glycine max* | Three Kingdom/Nam R | 6.5 | 4.2 |  |
| Charred *Glycine max* | Three Kingdom/Nam R | 6.7 | 4.9 |  |
| Charred *Glycine max* | Three Kingdom/Nam R | 6.6 | 3.9 |  |
| Charred *Glycine max* | Three Kingdom/Nam R | 6.8 | 4.5 |  |
| Charred *Glycine max* | Three Kingdom/Nam R | 6.8 | 5.2 |  |
| Charred *Glycine max* | Three Kingdom/Nam R | 6.3 | 4.2 |  |
| Charred *Glycine max* | Three Kingdom/Nam R | 7.0 | 3.5 |  |
| Charred *Glycine max* | Three Kingdom/Nam R | 6.7 | 3.7 |  |
| Charred *Glycine max* | Three Kingdom/Nam R | 4.8 | 2.6 |  |
| Charred *Glycine max* | Three Kingdom/Nam R | 5.9 | 4.1 |  |
| Charred *Glycine max* | Three Kingdom/Nam R | 6.3 | 4.1 |  |
| Charred *Glycine max* | Three Kingdom/Nam R | 6.6 | 4.0 |  |
| Charred *Glycine max* | Three Kingdom/Nam R | 6.2 | 4.0 |  |
| Charred *Glycine max* | Three Kingdom/Nam R | 7.7 | 4.9 | 1.9 |
| Charred *Glycine max* | Three Kingdom/Nam R | 7.0 | 3.6 | 2.6 |
| Charred *Glycine max* | Three Kingdom/Nam R | 6.5 | 4.8 |  |
| Charred *Glycine max* | Three Kingdom/Nam R | 6.4 | 3.5 |  |
| Charred *Glycine max* | Three Kingdom/Nam R | 5.2 | 3.3 |  |
| Charred *Glycine max* | Three Kingdom/Nam R | 4.5 | 2.4 |  |
| Charred *Glycine max* | Three Kingdom/Nam R | 7.1 | 4.5 |  |
| Charred *Glycine max* | Three Kingdom/Nam R | 6.1 | 4.6 |  |
| Charred *Glycine max* | Three Kingdom/Nam R | 6.8 | 4.6 |  |
| Charred *Glycine max* | Three Kingdom/Nam R | 7.1 | 4.6 |  |
| Charred *Glycine max* | Three Kingdom/Nam R | 6.8 | 4.5 |  |
| Charred *Glycine max* | Three Kingdom/Nam R | 6.5 |  |  |
| Charred *Glycine max* | Three Kingdom/Nam R | 5.4 |  |  |
| Charred *Glycine max* | Three Kingdom/Nam R | 6.5 | 4.0 |  |
| Charred *Glycine max* | Three Kingdom/Nam R | 6.4 | 4.3 |  |
| Charred *Glycine max* | Three Kingdom/Nam R | 6.6 |  |  |
| Charred *Glycine max* | Three Kingdom/Nam R | 5.0 | 3.7 |  |
| Charred *Glycine max* | Three Kingdom/Nam R | 6.8 | 3.2 |  |
| Charred *Glycine max* | Three Kingdom/Nam R | 6.6 | 3.6 |  |
| Charred *Glycine max* | Three Kingdom/Nam R | 6.8 | 3.4 |  |
| Charred *Glycine max* | Three Kingdom/Nam R | 7.1 |  |  |
| Charred *Glycine max* | Three Kingdom/Nam R | 6.8 | 4.0 |  |
| Charred *Glycine max* | Three Kingdom/Nam R | 7.3 | 4.0 |  |
| Charred *Glycine max* | Three Kingdom/Nam R | 6.4 | 5.0 |  |
| Charred *Glycine max* | Three Kingdom/Nam R | 5.6 | 4.5 |  |
| Charred *Glycine max* | Three Kingdom/Nam R | 6.2 | 3.9 |  |
| Charred *Glycine max* | Three Kingdom/Nam R |  | 3.5 |  |
| Charred *Glycine max* | Three Kingdom/Nam R |  | 4.8 |  |
| Charred *Glycine max* | Three Kingdom/Nam R | 6.1 | 4.0 |  |
